# Endless Forms: Within-Host Variation in the Structure of the West Nile Virus RNA Genome during Serial Passage in Bird Hosts

**DOI:** 10.1128/mSphere.00291-19

**Published:** 2019-06-26

**Authors:** Stacey L. P. Scroggs, Nathan D. Grubaugh, Johnny A. Sena, Anitha Sundararajan, Faye D. Schilkey, Darci R. Smith, Gregory D. Ebel, Kathryn A. Hanley

**Affiliations:** a Department of Biology, New Mexico State University, Las Cruces, New Mexico, USA; b Department of Microbiology, Immunology & Pathology, Colorado State University, Fort Collins, Colorado, USA; c National Center for Genome Resources, Santa Fe, New Mexico, USA; University of Pittsburgh School of Medicine

**Keywords:** RNA virus, West Nile virus, evolution, genetic diversity, natural selection, quasispecies, secondary structure, untranslated region

## Abstract

RNA viruses are infamous for their high rates of mutation, which produce swarms of genetic variants within individual hosts. To date, analyses of intrahost genetic diversity have focused on the primary genome sequence. However, virus phenotypes are shaped not only by primary sequence but also by the secondary structures into which this sequence folds. Such structures enable viral replication, translation, and binding of small RNAs, yet within-host variation at the structural level has not been adequately explored. We characterized the structural diversity of the 5′ untranslated region (UTR) of populations of West Nile virus (WNV) that had been subject to five serial passages in triplicate in each of three bird species. Viral genomes were sampled from host serum samples at each passage (*n* = 45 populations) and subjected to next-generation sequencing. For populations derived from passages 1, 3, and 5 (*n* = 9 populations), we predicted the impact of each mutation occurring at a frequency of ≥1% on the secondary structure of the 5′ UTR. As expected, mutations in double-stranded (DS) regions of the 5′ UTR stem structures caused structural changes of significantly greater magnitude than did mutations in single-stranded (SS) regions. Despite the greater impact of mutations in DS regions, mutations in DS and SS regions occurred at similar frequencies, with no evidence of enhanced selection against mutation in DS regions. In contrast, mutations in two regions that mediate genome cyclization and thereby regulate replication and translation, the 5′ cyclization sequence and the UAR flanking stem (UFS), were suppressed in all three hosts.

**IMPORTANCE** The enzymes that copy RNA genomes lack proofreading, and viruses that possess RNA genomes, such as West Nile virus, rapidly diversify into swarms of mutant lineages within a host. Intrahost variation of the primary genomic sequence of RNA viruses has been studied extensively because the extent of this variation shapes key virus phenotypes. However, RNA genomes also form complex secondary structures based on within-genome nucleotide complementarity, which are critical regulators of the cyclization of the virus genome that is necessary for efficient replication and translation. We sought to characterize variation in these secondary structures within populations of West Nile virus during serial passage in three bird species. Our study indicates that the intrahost population of West Nile virus is a diverse assortment of RNA secondary structures that should be considered in future analyses of intrahost viral diversity, but some regions that are critical for genome cyclization are conserved within hosts. Besides potential impacts on viral replication, structural diversity can influence the efficacy of small RNA antiviral therapies.

## Abstract (precis)

The enzymes that copy RNA genomes lack proofreading, and viruses that possess RNA genomes, such as West Nile virus, rapidly diversify into swarms of mutant lineages within a host. Intrahost variation of the primary genomic sequence of RNA viruses has been studied extensively because the extent of this variation shapes key virus phenotypes. However, RNA genomes also form complex secondary structures based on within-genome nucleotide complementarity, which are critical regulators of the cyclization of the virus genome that is necessary for efficient replication and translation. We sought to characterize variation in these secondary structures within populations of West Nile virus during serial passage in three bird species. Our study indicates that the intrahost population of West Nile virus is a diverse assortment of RNA secondary structures that should be considered in future analyses of intrahost viral diversity, but some regions that are critical for genome cyclization are conserved within hosts. Besides potential impacts on viral replication, structural diversity can influence the efficacy of small RNA antiviral therapies.

## INTRODUCTION

RNA viruses have exceptionally high mutation rates due to a lack of proofreading by viral RNA-dependent RNA polymerases (RdRp) (1, 2). As a consequence, RNA virus genomes rapidly diversify within individual hosts into a swarm of related mutant sequences (1–4). When this mutant swarm forms a unit upon which natural selection can act, it is termed a quasispecies (2, 5). Intrahost genetic diversity can shape viral phenotypes, including virulence and pathogenicity (6–15). To date, the characterization of virus genetic diversity has focused on quantifying variation in the linear RNA nucleotide sequence (6, 16–29). However, RNA genomes fold into complex secondary and tertiary structures, many located in untranslated regions (UTRs), which have essential functions in the viral life cycle (30–35). Alteration of the secondary structure *per se* has been shown to affect viral fitness (36–39). Additionally, for many RNA viruses, efficient genome replication and translation depend on long-range binding of sequences in the 5′ and 3′ UTRs that circularize the linear viral genome (40, 41).

Secondary structures in RNA virus genomes are generally highly conserved among consensus sequences of viruses isolated from separate hosts, reflecting their critical roles in the virus life cycle (42–47). The strength of selection is such that during passaging of viruses with altered secondary structures, compensatory mutations often arise that partially or completely reconstitute the wild-type structure (see, e.g., references 29 and 47). However, the vast majority of *in silico* analyses of viral genome structures to date (see, e.g., references 45 and 47–50) have relied on consensus sequences which do not reveal intrahost variation. An exception is a recent study by Ziv et al. (28), in which, using a novel cross-linking technique, at least five major alternative conformations of the Zika virus (ZIKV) genome were detected *in vivo*. Additionally, de Borba et al. (29) identified 10 single-nucleotide variants within secondary structures in the dengue virus (DENV) 3′ UTR that were under positive selection during replication in mosquitoes. Characterizing intrahost variation in RNA virus genome structure and the forces that shape it will provide deeper insight into the effects of population diversity on viral phenotypes, guide investigations of quasispecies dynamics, and reveal optimal targets for small RNA antiviral therapies (5, 51, 52).

**FIG 1:**
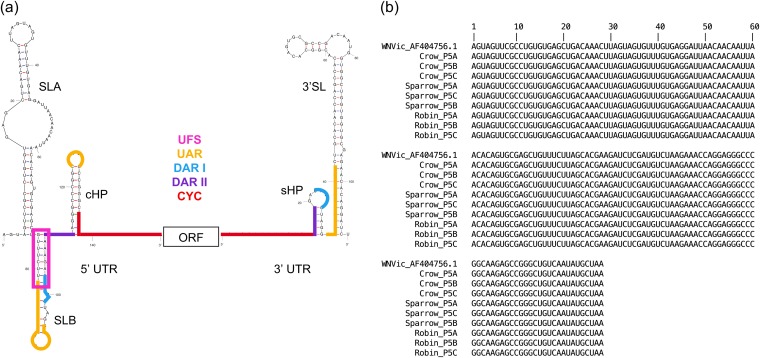
(a) Representation of the WNV structural elements and binding sites of the 5′ and 3′ UTRs flanking the open reading frame (ORF). To initiate replication once cyclization has occurred, SLA binds to and directs the RdRp to the 3′ UTR (56–58). SLB contains the start codon for translation (55, 62), and cHP is a translation enhancer (67). The structure was generated using mFold online version 4.7 (74). Binding sites were identified by Brinton (62). (b) Alignment of the consensus sequences of the first 150 nucleotides of the WNV genome, via Geneious (105), shows that sequences of WNV after five passages in designated hosts are 100% identical with each other and with parental wild-type WNV infectious clone 3356 (WNVic).

In the current study, we tracked structural variation in the West Nile virus 5′ UTR over the course of serial passage in avian hosts. West Nile virus (WNV; genus Flavivirus) possesses a positive-sense RNA genome flanked by 5′ and 3′ UTRs (Fig. 1a). To facilitate replication and translation, secondary structures within the 5′ and 3′ UTRs of flaviviruses position the specific sequences (here referred to as binding sites) for long-range binding to achieve genome cyclization and also recruit and bind host and viral factors for replication and translation (43, 53–58). The linear and cyclized conformations of the genome occur at an approximately 1:1 ratio (59, 60). The transition between the linear and circular forms of the genome is mediated by the 5′ upstream of start codon (UAR) flanking stem (UFS) structure (61). The 5′ UTR was chosen for our analysis because it contains well-defined secondary structures of known function (Fig. 1a), stem-loops A and B (SLA and SLB, respectively), the UFS, and the capsid-coding region hairpin (cHP) (30, 62). Moreover, at 96 nucleotides, the 5′ UTR is small enough to be encompassed by a single 150-nucleotide Illumina sequencing read.

WNV is maintained in birds and Culex mosquitoes and occasionally spills over to cause disease in horses and humans (63). In North America, the American crow, house sparrow, and American robin are known to propagate WNV outbreaks. These three species exhibit substantial differences in viremia and mortality. To explore host-specific impacts on the evolutionary dynamics of diversification of the open reading frame (ORF) sequence, WNV was previously passaged five times in triplicate in each of the three bird species, viral RNA was isolated from each infected bird, and viral genomes were sequenced (64). This study found that, in the ORF, all WNV populations were subject to strong purifying selection, but unique mutations and defective genomes were most frequent in virus passaged in crows, followed by those passaged in sparrows and then robins (64).

Here, we first sought to characterize structural diversity in each population by predicting the structure of the 5′ UTR carrying each mutation that occurred at a frequency of ≥1%, using two different structure prediction algorithms. We predicted only the secondary structures of the linear genome, because neither algorithm is capable of predicting pseudoknots and because we did not have complementary data on mutations in the 3′ UTR. While the WNV genome has three known pseudoknots, located in the capsid gene, NS1/2A genes, and the 3′ UTR (62), no pseudoknots have been identified in the 5′ UTR. Moreover, multiple studies have shown that mutations that distort the configuration of the secondary structures are deleterious for the fitness of WNV, as well as other flaviviruses (57, 58, 61, 65–68).

We then used our predicted structures to test the hypothesis that purifying selection acts to preserve secondary structures during replication within hosts, as it does during transmission between hosts. This hypothesis generated several testable predictions. First, it predicts that mutations with a disproportionately large impact on the secondary structure will be relatively infrequent in the population. Studies of HIV (45, 46, 69–72) and hepatitis C virus (73) indicate that changes in nucleotides in double-stranded (DS) regions of RNA secondary structures are more likely to induce structural changes than are changes in single-stranded (SS) regions. Moreover, using consensus sequences of HIV, Assis (45) showed that selection against mutations that break the base pairings that produce the DS structure was 2.7 times stronger than selection against mutations that did not cause such breakage. Consistent with these studies, we also detected a disproportionate impact of variation in DS regions on predicted WNV 5′ UTRs in the current study. We therefore predicted that variants in DS regions would be less common than would variants in SS regions. Additionally, we predicted that variants producing major structural changes would be less common than those producing minor changes. Finally, we predicted that variants in sequences or structures that enable or regulate the formation of the circularized genome, namely, binding sites and the UFS riboswitch (Fig. 1a), would be less common than variants outside the binding sites. While our data did not support the first two predictions, it did reveal a suppression of variation in the cyclization sequence (CYC) binding site and the UFS riboswitch for all three avian hosts.

## RESULTS

### Overview of WNV 5′ UTR variants.

In order to assess the impact of different avian hosts on WNV diversity and fitness, WNV infectious clone 3356 (WNVic) was passaged five times in each of three different bird species in triplicate (64). Each viral population was then sequenced using an Illumina platform. Data from the open reading frame (ORF) were analyzed previously (64). In the current study, we analyzed the secondary structure of the 5′ UTR in populations from the first, third, and fifth passages in order to capture rapid, intermediate, and gradual selection, respectively. The total number of 5′ UTR paired sequenced reads per bird and replicate for all passages varied from 3,198 (robin passage 5, replicate B) to 3,155,764 (sparrow passage 3, replicate B). The average number of paired reads by bird across all 3 passages and replicates were 726,362 for crows, 868,899 for sparrows, and 522,048 for robins.

We analyzed only variants occurring at a frequency of ≥1.0%. This sample included a total of 133 variants from passage 1 (15 crow, 30 sparrow, and 88 robin), 178 variants from passage 3 (62 crow, 62 sparrow, and 54 robin), and 192 variants from passage 5 (44 crow, 74 sparrow, and 74 robin). A summary file of variant frequencies was used to generate this set of variants; consequently, linkages of variants within haplotypes could not be determined. Most of these variants occurred at frequencies that ranged from 1.0 to 38.2% (Table 1), but at passage 3, crow replicate C and sparrow replicate B contained two and five mutations, respectively, that reached consensus (i.e., occurred in more than 50% of the reads) but not fixation. However, high frequency mutations were not detected by passage 5, and the consensus sequences of virus populations from each replicate (A, B, and C) in each of the three bird hosts show 100% identity of the 5′ UTR sequence with each other and with parental WNVic (Fig. 1b).

**TABLE 1:** Summary of paired reads and variants with frequencies of ≥1.0% from the 5′ UTR

Passage no.	Replicate	Total no. of 5′ UTR reads	Range in frequency of variants occurring at ≥1.0 (%)	No. of unique variants ≥1.0%
1	Crow			
	A	1,105,842	1.0	1
	B	1,003,674	1.1–1.8	6
	C	1,429,252	1.2–2.1	8
	Sparrow			
	A	1,163,411	1.0–16.4	3
	B	1,146,719	2.3–13.5	5
	C	366,481	1.0–2.6	22
	Robin			
	A	70,153	1.0–6.5	39
	B	1,462,416	1.0–4.4	26
	C	93,296	1.0–3.9	23
3	Crow			
	A	898,274	1.0–4.2	30
	B	821,125	1.0–3.6	27
	C	1,189,599	1.0–52.3	5
	Sparrow			
	A	728,521	1.5–14.4	9
	B	3,155,764	1.0–65.7	18
	C	1,195,437	1.0–17.1	35
	Robin			
	A	622,342	1.0–1.9	8
	B	2,142,138	1.0–3.1	26
	C	541,686	1.0–2.9	20
5	Crow			
	A	29,162	1.1–1.3	4
	B	29,682	1.0–3.9	24
	C	30,649	1.0–6.4	27
	Sparrow			
	A	23,897	1.0–2.3	19
	B	14,837	1.0–13.0	36
	C	25,027	1.0–6.1	22
	Robin			
	A	23,898	1.0–38.2	35
	B	3,198	1.0–3.0	25
	C	9,308	1.0–2.9	24

To assess the impact of each variant, the corresponding wild-type (wt) nucleotide was replaced *in silico* with the mutated nucleotide within a sequence encompassing the first 150 nucleotides of the wt WNVic sequence. The secondary structure for all mutated sequences was then predicted using the mFold application version 4.7 (http://unafold.rna.albany.edu/ [74]). The resulting structures with the lowest Gibbs free energy were compared to the wt structure, and changes between the two were categorized as major if six or more changes in strandedness occurred between the wt and the mutated structure (e.g., SS to DS or DS to SS), minor if fewer than six such changes occurred, and none if no structure change was predicted. As our categorization of major and minor was arbitrary, the analyses that compared major versus minor versus no structure change were repeated to compare any structure change versus no structure change. After the final passage, 21 variants occurred in all three hosts, and 8 variants occurred in two hosts (Table 2). Of the variants that occurred in all three species, 57.1% caused a minor structure change and 42.9% caused no structure change. Of the variants that occurred in two hosts, 50% resulted in minor structure change, while 37.5% resulted in a major structure change, and the remaining 12.5% did not alter the structure.

**TABLE 2:** Twenty-nine variants from passage 5 that were found in more than one avian host

Variant	Structure change	Passage 5 replicate(s)		
		Crow	Sparrow	Robin
A124T	None	B, C	A, B, C	A, B, C
A125G	None	B, C	A, B, C	A, B, C
A127C	None	B, C	A, B, C	A, B, C
A127T	None	B, C	A, B, C	A, B, C
C118T	None	B, C	A, B, C	A, B, C
C120G	Minor	B, C	A, B, C	A, B, C
C123T	None	B, C	A, B, C	A, B, C
C130A	Minor	B, C	A, B, C	A, B, C
G116A	None	B, C	A, B, C	A, B, C
G121T	Minor	B, C	A, B, C	A, B, C
G122A	Minor	B, C	A, B, C	A, B, C
G126T	None	B, C	A, B, C	A, B, C
G128C	Minor	B, C	A, B, C	A, B, C
G131T	Minor	B, C	A, B, C	A, B, C
G132C	Minor	B, C	A, B, C	A, B, C
G132T	Minor	B, C	A, B, C	A, B, C
G117A	Minor	B, C	B	A, C
G115A	None	B, C	B	A
A114C	Minor	C	B	A, C
G113A	Minor	C	B	A, C
C123G	Minor	C	B	A
A34T	Major	A, B	B	
A50G	Major	C	B	
A26T	Major		B	A
G2A	None		C	A
G121A	Minor		B	B
G131A	Minor		B	A
T3C	Minor		C	A, B
T7C	Minor		A, C	B, C

### Overview of WNV 5′ UTR structures.

To predict the secondary structure, we initially folded all variant 5′ UTR sequences using mFold with the temperature set to 37°C. All resulting unique 5′ UTR structures (*n* = 80) are shown in Table S1 in the supplemental material; 41.8% of all variants resulted in no change, 49.5% resulted in a minor change, and 8.6% resulted in a major change. We next tested whether our choice of mFold had a large impact on our predicted structures by randomly selecting 20% of the unique structures (*n* = 16) and predicting their structures in RNAstructure version 6.0.1 (https://rna.urmc.rochester.edu/RNAstructureWeb/) at 37°C (Table S2). Eighty-one percent of the outputs from mFold and RNAstructure were identical; of those that differed, none shifted from no change to major change. The distribution of major, minor, or no structural changes did not differ significantly among the structures predicted in mFold and RNAstructure (χ^2^ = 1.7, *df* = 2, *P* = 0.43). Next, we tested whether folding sequences at 40°C, the average body temperature of birds, rather than at 37°C affected the structure predictions. The same 16 structures were refolded in RNAstructure version 6.0.1 at 40°C (Table S2); 100% were identical in structure classification (e.g., major, minor, or no change from the wild type). We also tested the potential effect of high fever in crows on the predicted secondary structures of passage 5 variants and found that 96.0% of the structures that folded at 37°C showed the same structure change classification when the variants were folded at 42°C; there was also not a significant difference in the distribution of major, minor, and no structure changes between the structures predicted at 37°C and 42°C (χ^2^ = 0.17, *df* = 2, *P* = 0.92).

10.1128/mSphere.00291-19.1 TABLE S1 5′ UTR structures. Download Table S1, DOCX file, 2.1 MB. Copyright © 2019 Scroggs et al. 2019 Scroggs et al. This content is distributed under the terms of the Creative Commons Attribution 4.0 International license.

10.1128/mSphere.00291-19.2 TABLE S2 Structure comparison from mFold and RNAstructure at 37°C and 40°C. Download Table S2, DOCX file, 2.0 MB. Copyright © 2019 Scroggs et al. 2019 Scroggs et al. This content is distributed under the terms of the Creative Commons Attribution 4.0 International license.

To assess the impact of selecting the first (lowest free energy) structure predicted by mFold rather than the second (i.e., next lowest free energy) structure on our analysis, we compared the first and second predicted structures from mFold for the 16 mutations described above and found that the prediction of major, minor, and no structure change was identical for 15 out of the 16 mutations (Table S3).

10.1128/mSphere.00291-19.3 TABLE S3 Comparison of first and second predicted structures and Δ*G* values (kcal/mol) by mFold. Download Table S3, DOCX file, 0.9 MB. Copyright © 2019 Scroggs et al. 2019 Scroggs et al. This content is distributed under the terms of the Creative Commons Attribution 4.0 International license.

### Impact of SS or DS variant location on structure change.

**FIG 2:**
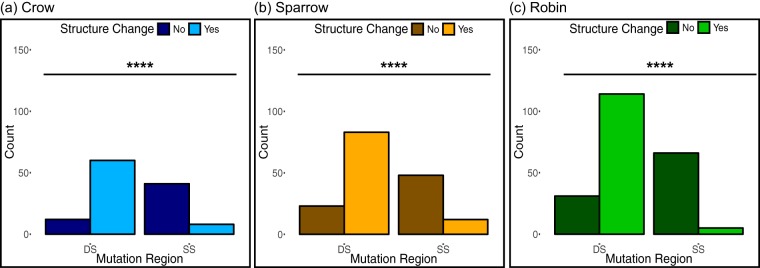
(a to c) Variants in DS regions of the WNV 5′ UTR sequence were more likely to cause predicted structure changes than were variants in SS regions in virus passaged in crows (a), sparrows (b), and robins (c). The tendencies of variants from wild-type sequence in DS and SS regions to cause any change in secondary structure were compared using Fisher’s exact test for virus populations pooled across passages 1, 3, and 5 for each bird species (*n* = 121 variants for crow, *n* = 166 for sparrow, and *n* = 216 for robin). ****, *P* < 0.0001.

**FIG 3:**
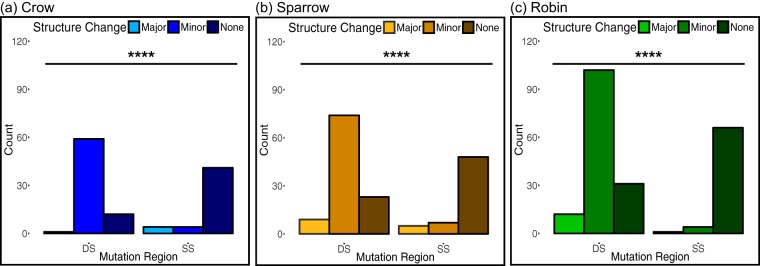
Variants in DS regions of the WNV 5′ UTR were more likely to cause minor structure change than were variants in SS regions, while variants in the DS and SS regions were equally likely to cause major structure change. (a to c) Variants in SS regions were more likely to cause no structure change than were variants in DS regions in viruses passaged in crows (a), sparrows (b), and robins (c). The degrees of predicted structure change as a result of variants in SS or DS regions were compared using Fisher’s exact test for samples from each bird species pooled across passages 1, 3, and 5 (*n* = 121 variants for crow, *n* = 166 for sparrow, and *n* = 216 for robin). ****, *P* < 0.0001.

We first tested whether variation from the wild-type sequence in DS regions would be more likely to cause predicted structure change than would variation in SS regions. This assumption was borne out for all three bird-passaged WNV populations, as shown in Fig. 2a to c. Next, we evaluated the degree of predicted structure change and found that variants in DS regions were significantly more likely to cause minor structure changes than were variants in SS regions, while variants in SS regions tended not to alter structure (Fig. 3a to c). However, variants in DS and SS regions were equally likely to cause major structure change.

### Distribution of variants in DS and SS regions.

**FIG 4:**
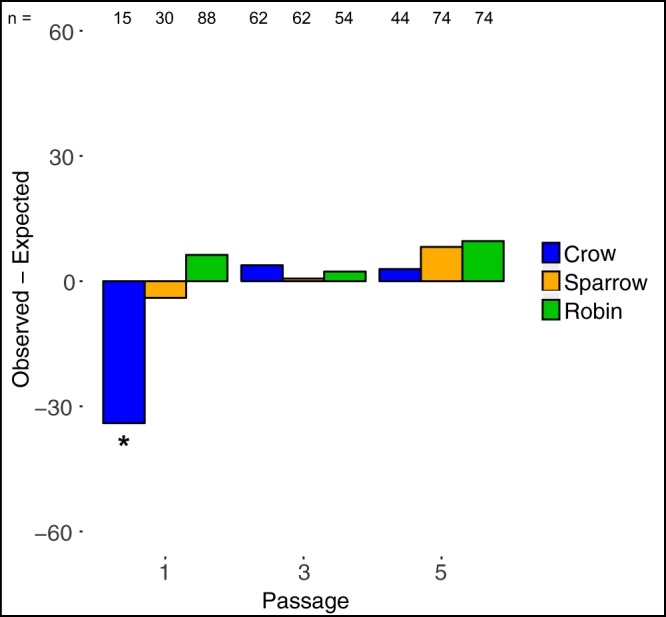
Variants were not less common in DS regions than expected in the WNV 5′ UTR for virus passaged in crows, sparrows, and robins, with the exception of the variants for WNV passaged in crows once, which were more likely to occur in SS regions. Positive values indicate more variants than expected in a DS region, and negative values indicate more variants than expected in an SS region. Using a chi-square test with Bonferroni’s correction for multiple comparisons, only passage 1 variants from crow-passaged WNV differed from the expected (χ^2^ = 7.3, *df*: 1, *P* = 0.007, adjusted *P* = 0.02); see Table S4 for complete statistics. *, adjusted *P* < 0.05.

To determine whether the disproportionate impact of variation in DS regions led to selection for the suppression of such variants, we tested whether variants in DS regions occurred less frequently than expected based on the percentage of nucleotides in DS regions in the wild-type WNV 5′ UTR structure. The distribution of variants in the DS and SS regions of the linear genome at each passage was compared to the expected distribution of the first 150 nucleotides (nt) of wild-type WNV, which is 60.7% DS and 39.3% SS. When stratified by passage, WNV populations showed no significant variation from the expected distribution of variants occurring in DS and SS regions after correction for multiple comparisons, as shown in Fig. 4 and Table S4. Moreover, there was no indication of a decrease or increase in the percentage of variants occurring in DS regions over the course of the five passages, with the exception of viruses passaged once in crows, wherein more variants than expected occurred in SS regions (adjusted *P* = 0.02) (Fig. 4 and Table S4). As an additional analysis of selection on mutations in DS versus SS regions, we followed the approach used by Assis (45) in which an exact binomial test was used to detect differences in mutation saturation levels between DS and SS sites. In crows, DS and SS sites were equally saturated with mutations, except for passage 1, which has 6.1% saturation at SS nucleotides compared to 1.5% for DS nucleotides (adjusted *P* = 0.002; see Table 3 for full statistics). The saturations of mutations at DS and SS nucleotides in WNV passaged in sparrows and robins were most often equal, but the saturation of mutations at DS nucleotides was greater than that at SS nucleotides at passages 1 and 5 for robins and passage 5 for sparrows (adjusted *P* < 0.05 for robin passages 1 and 5 and sparrow passage 5; see Table 3 for full statistics). The average difference was an increase of 6.3% in saturation at DS sites.

**TABLE 3:** Saturation of mutations in DS and SS sites

Passage no.	Bird	No. of mutations:				Saturation (%) of:		*P* value	Adjusted *P* value^*a*^
		In DS regions	In SS regions	Per DS site	Per SS site	DS sites	SS sites		
1	Crow	4	11	0.04	0.18	1.5	6.1	0.0005	0.002
	Sparrow	17	13	0.19	0.22	6.3	7.2	0.6	1.00
	Robin	59	29	0.66	0.48	21.9	16.1	0.01	0.03
3	Crow	40	22	0.44	0.37	14.8	12.2	0.2	0.57
	Sparrow	38	24	0.42	0.40	14.1	13.3	0.7	1.00
	Robin	34	20	0.38	0.33	12.6	11.1	0.4	1.00
5	Crow	28	16	0.80	0.82	10.4	8.9	0.4	1.00
	Sparrow	51	23	0.57	0.38	18.9	12.8	0.004	0.01
	Robin	52	22	0.58	0.37	19.3	12.2	0.0007	0.002

a Adjusted with Bonferroni’s correction for multiple comparisons.

10.1128/mSphere.00291-19.4 TABLE S4 χ^2^ test results for differences between expected and observed distributions of DS and SS mutations from Fig. 4. Download Table S4, DOCX file, 0.01 MB. Copyright © 2019 Scroggs et al. 2019 Scroggs et al. This content is distributed under the terms of the Creative Commons Attribution 4.0 International license.

### Frequency of predicted structure change in the WNV 5′ UTR among different host species.

Grubaugh et al. (64) reported significant differences in WNV fitness and in the distribution and frequency of mutations in virus populations passaged in different hosts. However, in the current study, there was no difference among species in the total number of variants that resulted in predicted structure change (e.g., major and minor structure change combined versus no structure change; Kruskal-Wallis χ^2^ = 2, *df* = 2, *P* = 0.37) or even a correspondence of the ranking of the total number of change-driving variants (crow > robin > sparrow) with the relative fitness reported by Grubaugh et al. (64) (robin > sparrow > crow). The same patterns held for variants that caused major structure changes (robin > crow > sparrow; Kruskal-Wallis χ^2^ = 2, *df* = 2, *P* = 0.37). The frequency of variants in DS regions, irrespective of predicted structure change, did not differ among hosts (59.5% variants in DS regions for crows, 53.9% for sparrows, and 67.1% for robins; χ^2^ = 2.0, *df* = 2, *P* = 0.4).

### Viral variants in the sequences and structures that enable and regulate genome circularization.

As the WNV binding sites and the UFS together regulate transitions between the linear and circular genome, we predicted that selection should act to purge variation within these sites, in particular relative to the remainder of the 5′ UTR sequence. To test this prediction, we compared the proportion of variants within and outside specific elements to the proportion of nucleotides within and outside these elements to assess whether variants within them occur more or less often than expected by chance. Additionally, we used an exact binomial test (45) to compare mutation saturation levels within and outside the specific elements.

**FIG 5:**
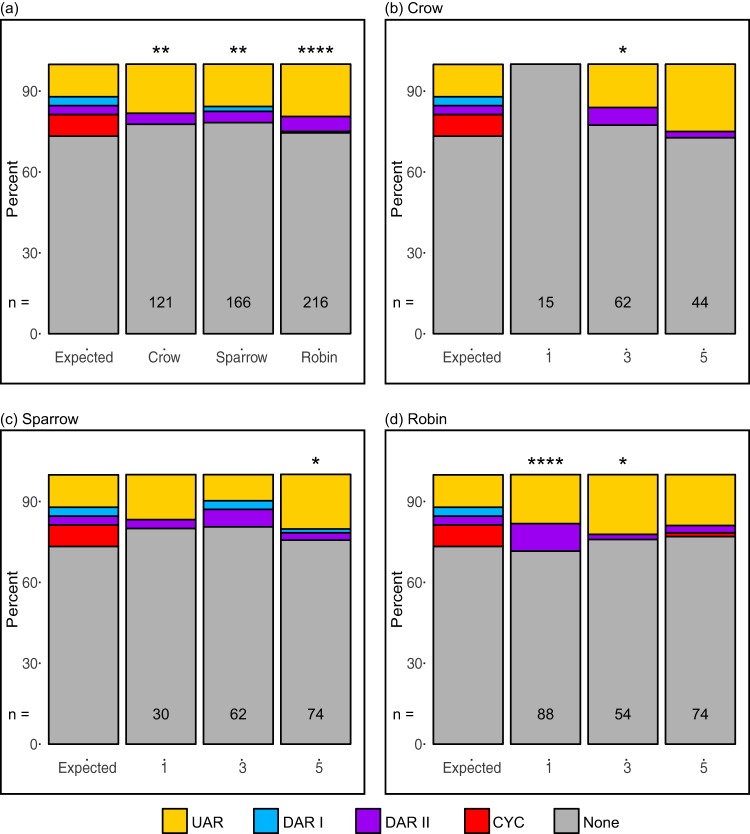
The frequency of variants within designated binding sites in the WNV 5′ UTR differed from the expected distribution. (a) When the variants were pooled by passage (numbers 1, 3, and 5 on the bottom of each graph), all bird-passaged WNV populations had fewer variants than expected in the CYC binding site. (b to d) Variants located in binding site sequences by passage for viruses passaged in crows (b), sparrows (c), and robins (d) were less common in all binding sites except the UAR. Full statistics from χ^2^ tests are in Table S5. The UFS riboswitch was also analyzed, and results are described in the text; however, as this sequence overlaps binding sequences, it could not be illustrated in this figure. *, *P* < 0.05; **, *P* < 0.01; ****, *P* < 0.0001.

Figure 5a depicts the expected distribution of nucleotides within and outside the binding sites (UAR, DAR I, DAR II, and CYC), as well as the host-specific distributions of variants in these regions, pooled across passages 1, 3, and 5. Fewer variants occurred in the CYC binding site sequence than expected in each of the three bird species. The frequency of variants in the DAR II binding site was not significantly different from the expected frequency in any bird species.

10.1128/mSphere.00291-19.5 TABLE S5 χ^2^ test results for differences between expected and observed mutations within and outside binding sites from Fig. 5b to d. Download Table S5, DOCX file, 0.01 MB. Copyright © 2019 Scroggs et al. 2019 Scroggs et al. This content is distributed under the terms of the Creative Commons Attribution 4.0 International license.

When stratified by passage and host species (Fig. 5b to d and Table S5), variants in the CYC binding site sequence remained less common than expected, although many of the differences were no longer significant due to small sample size. For all three hosts, at passage 1, variants were less common in the CYC binding site, although the difference was only significant for the robin-passaged virus and were more common in the UAR binding site, except for the 15 crow-passaged variants, which were all located outside a binding site. By passage 3, variants were still less common in the CYC binding site, but the difference was only significant for crow- and robin-passaged viruses. At passage 5, variants were less common in the DAR I, DAR II, and CYC binding sites than expected; however, they were more common in the UAR binding site across all host species; these differences were only significant for sparrow-passaged virus.

A binomial analysis of mutation saturation within and outside binding sites relative to other regions of the 5′ UTR revealed that, with the exception of the UAR in robin-passaged virus, when significant differences were detected, they reflected lower mutation saturation in the binding sites (Table 4). Mutation saturation inside the CYC binding site was significantly lower than that outside the binding site for virus passaged in each of the three host species.

**TABLE 4:** Saturation of mutations within and outside binding sites and the UFS element

Bird	Binding site^*a*^	No. of mutations:				Saturation (%) of:		*P* value	Adjusted *P* value^*b*^
		Inside	Outside	Per inside site	Per outside site	Inside sites	Outside sites		
Crow	UAR	20	94	1.11	0.29	37.04	28.48	0.18	0.70
	DAR I	0	94	0.00	0.29	0.00	28.48	0.009	0.04
	DAR II	5	94	1.00	0.29	33.33	28.48	0.78	1.00
	CYC	0	94	0.00	0.29	0.00	28.48	7.98E−06	0
	All BS	25	94	0.63	0.29	20.83	28.48	0.07	NA
	UFS	0	121	0.00	0.90	0.00	30.10	7.30E−08	NA
Sparrow	UAR	26	130	1.44	0.29	48.15	39.39	0.21	0.84
	DAR I	3	130	0.60	0.29	20.00	39.39	0.19	0.74
	DAR II	7	130	1.40	0.29	46.67	39.39	0.60	1.00
	CYC	0	130	0.00	0.29	0.00	39.39	2.47E−08	0
	All BS	36	130	0.90	0.29	30.00	39.39	0.04	NA
	UFS	0	166	0.00	1.24	0.00	41.29	1.18E−11	NA
Robin	UAR	42	161	2.33	0.29	77.78	48.79	1.60E−05	0
	DAR I	0	161	0.00	0.29	0.00	48.79	6.48E−05	0
	DAR II	12	161	2.40	0.29	80.00	48.79	1.88E−02	0.08
	CYC	1	161	0.08	0.29	2.78	48.79	1.45E−09	0
	All BS	55	161	1.38	0.29	45.83	48.79	0.52	NA
	UFS	1	215	0.06	1.60	2.08	53.48	6.26E−15	NA

a BS, binding sites.

b Adjusted with Bonferroni’s correction for multiple comparisons. NA, not applicable.

In the UFS, only a single mutation was detected from passages 1, 3, and 5 at a frequency over 1.0%. This mutation was identified from virus passaged once in robins at a frequency of 1.2% and did not alter the structure of the 5′ UTR. The difference in the distributions of mutations inside and outside the UFS compared to the expected distribution was significantly different for all hosts pooled by each passage (*P* < 0.0001 for all three). The saturation of mutations in the UFS site of virus passaged in robins was significantly lower, at 2.1% compared to 53.5% mutation saturation outside the UFS site (*P* = 6.26E−15, Table 4).

### Sensitivity of structure of 3′ and 5′ UTR cyclization binding sites to nucleotide variation.

**FIG 6:**
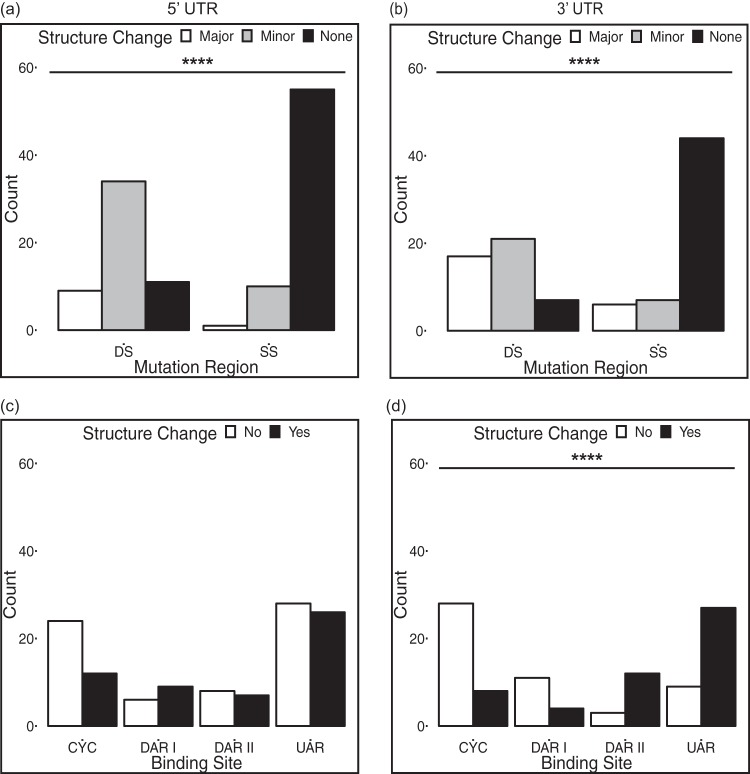
Analysis of the impact of all possible variants within 5′ and 3′ UTR binding sites on predicted structure. (a and b) Variants in DS regions in both the 5′ UTR and the 3′ UTR tend to cause structure change (minor and major), while variants in SS regions do not, as detected by Fisher’s exact tests. (c and d) In the 5′ UTR, variants in each of four binding sites are equally likely to result in structure change or no structure change; in the 3′ UTR, variants in different binding sites differ significantly in their tendency to cause structure change, as detected by Fisher’s exact tests. ****, *P* < 0.0001.

The sequencing coverage of the WNV 3′ UTR in this study was not adequate for our analysis because the reads did not consistently cover to the 3′ terminus of the 3′ UTR. To understand the potential impact of variants in the 3′ UTR binding sites on structure, we systematically altered the sequences of the 3′ binding sites of the wild-type WNV genome *in silico* (UAR, nt 10951 to 10962 [83% DS]; DAR I, nt 10942 to 10946 [20% DS]; DAR II, nt 10935 to 10939 [100% DS], and CYC, nt 10923 to 10934 [0% DS]) to reflect all three possible alternative nucleotides at each position. For example, if the original nucleotide was an A, the three mutated sequences contained a C, G, or T at that position. This analysis was implemented on the 5′ UTR binding sites as well (UAR, nt 82 to 95 and 124 to 127 [55% DS]; DAR I, nt 99 to 103 [80% DS]; DAR II, nt 109 to 113 [40% DS]; and CYC, nt 135 to 146 [25% DS]). The sequences were folded using mFold. For both the 5′ and 3′ UTR, variants in DS regions caused more structure change than did variants in SS regions, which most often did not cause structure change (Fig. 6a and b).

When stratified by binding site, structure change and no structure change were equally likely in response to variants at each of the four binding sites in the 5′ UTR (Fig. 6c, Fisher’s exact *P* = 0.3). In the 3′ UTR, variants in the DAR II and UAR cause structure changes more often than did variants that occur in the CYC and DAR I binding sites (Fig. 6d).

## DISCUSSION

This study characterized intrahost variation in the secondary structures of the untranslated regions of the WNV genome. Specifically, we tested the hypothesis that purifying selection, which acts to conserve secondary structures of many viruses during transmission between hosts (42, 43, 45, 47, 75), would also act to conserve secondary structures of the linear genome as well as binding sites and structures that promote and regulate formation of the circularized form of the genome. We focused on the WNV 5′ UTR, which folds into several structures that are critical for virus fitness (57, 58, 65–68) and are highly conserved among different WNV isolates (55, 62) and across the mosquito-borne flaviviruses generally (30, 44). Further, we leveraged an experimental evolution data set to track variation and the overall evolution of WNV genome structures across serial passages in different avian hosts (64).

Using *in silico* structure predictions, we first demonstrated that, as in other viruses (42, 45, 76, 77), nucleotide variation in DS regions of the 5′ UTR was more likely to alter the folded structure of the 5′ UTR than was variation in SS regions. Unfortunately, due to insufficient read coverage, we were not able to perform the same analyses of variant distribution in the WNV 3′ UTR. To overcome this limitation, we instead analyzed the impacts of all possible variants within the four binding sites in both the 5′ UTR and 3′ UTR known to mediate genome cyclization by binding to each other. This analysis also demonstrated that the variants in DS regions caused changes in predicted structure significantly more often than did variants in SS regions.

While the majority of mutant genomes are likely inviable, they are not irrelevant because these genomes provide the pool of variability upon which selection can act when the environment of the virus changes due, for example, to stimulation of the immune response, a host shift, or drug treatment. Despite the inordinately large impact of variants in DS regions on predicted secondary structure, such variants were detected as frequently as expected by chance, contradicting our initial prediction that they would be subject to detectable purifying selection. Indeed, an analysis of mutation saturation revealed that three out of nine viral populations were more saturated with mutations in DS sites than in SS sites. Moreover, there was no evidence that the frequency of variants resulting in predicted structural changes, irrespective of DS or SS location, changed in a concerted way over the course of experimental passage, or covaried with fitness, as measured by Grubaugh et al. (64). In contrast, intrahost purifying selection on the amino acid sequence has been documented for WNV (17, 78–83), as well as arthropod-borne viruses, like DENV and bluetongue virus, and for directly transmitted viruses, like HIV and hepatitis C virus (HCV) (84–89). Grubaugh et al. (64) assessed intrahost selection on the ORF of the WNV populations analyzed in this study and found that the proportion of variants that resulted in nonsynonymous changes decreased after five passages (0.7 at input to 0.18 to 0.25 at passage 5), although the frequency of unique intrahost single nucleotide variants in the ORF increased (64). Grubaugh et al. (64) detected lower nucleotide variation in the ORF at passage 5 than we did in the 5′ UTR, yet they were able to identify selection across the ORF. This indicates that the detection of selection in the 5′ UTR is possible, although the region is smaller and could experience more stochastic variation. Thus, while purifying selection on the amino acid sequence of within-host WNV populations was detectable after five passages in the selected bird species (64), selection to preserve UTR secondary structures in the same populations was not.

However, our study did reveal evidence of purifying selection on the sequence of the 5′ CYC binding site and the UFS structural element. In all three bird species, fewer variants occurred in the CYC binding site sequence and the UFS than was expected. The 5′ CYC is the first binding site to bind the 3′ UTR during cyclization and is thus considered the most important site for replication (30), and the UFS is critical for viral replication and genome cyclization (61). Viral replication is suppressed when mutations that break the UFS double bonds are introduced but is partially restored with compensatory mutations (61). Point mutations in the WNV 5′ CYC binding site have been shown to abolish 5′ and 3′ genomic interactions (54) resulting in decreased replication and fitness (90, 91). Mutations in the 5′ CYC site may have prevented replication or may, by preventing cyclization, have accelerated genome degradation by exoribonucleases (92). Either effect may enhance the strength of selection against variants in the 5′ CYC binding site relative to other cyclization sites. Previous analyses of mutations in the 5′ CYC (54, 90, 91) did not include any of the variants identified in our study, so the impact of these specific variants on cyclization is not yet known. Recently, human microRNA required for viral propagation was found to bind the 5′ CYC of the ZIKV genome (28), demonstrating the importance of the 5′ CYC beyond intragenomic long-range viral RNA-RNA binding.

We acknowledge three important caveats to the conclusions above. First, all of our inferences are based on predicted structures, which are not a perfect representation of the actual structures adopted by the virus genome (see, e.g., reference 93). Because we sought to analyze individual variants rather than consensus populations, we were necessarily limited to analyzing predicted structures. In particular, the programs we used to predict structure cannot predict pseudoknots, the presence of which could certainly influence the fitness impacts of variants. While the WNV pseudoknot in the capsid gene, called the downstream cyclization sequence (dCS), spans nt 171 to 438 and is located near the 5′ UTR, there are no documented pseudoknots within the 5′ UTR (94). Recent empirical structural characterizations of the complete genomes of DENV (95) and ZIKV (28) confirmed the presence of the dCS pseudoknot and did not identify any pseudoknots within the 5′ UTR. While unlikely, future analyses may reveal WNV-specific pseudoknots not previously identified, which would then prompt us to reexamine our findings. Additionally, our analysis focused on the structures present in the linear genome, which can be different than the cyclized genome. For example, in the cyclized ZIKV genome, cHP remains mostly intact, but SLB opens completely and binds to the 3′ UTR (28).

Second, because folding and analyzing each structure were laborious, we analyzed only the variants that occurred at a frequency of ≥1.0%, an arbitrary cutoff.

Third, we focused only on avian hosts, but WNV undergoes an alternating cycle of replication in hosts and vectors, and the diversity of the WNV population depends whether the virus is replicating in host or vector. In the avian host, strong purifying pressure restricts genetic diversity, whereas this selection pressure is relaxed in the mosquito vector, resulting in increased diversity (17, 78, 79, 81, 96, 97). Thus, structural diversification may follow different patterns in the mosquito vector relative to the patterns found in the current study.

The structural variation detected in this study could influence the efficacy of antisense oligomer (98, 99) and small interfering RNA (siRNA) antiviral therapies (100) against WNV and contribute to the evolution of resistance to these agents. Many antisense agents are designed to bind sequences in the UTRs, and their binding relies upon perfect complementarity to the target sequence (101, 102). As appealing as antisense antiviral therapies may be, they must be considered in the context of the rapidly evolving virus populations. Using the same WNV infectious clone as that used in this study, Deas et al. (101) demonstrated that translation of WNV was suppressed by a phosphorodiamidate morpholino oligomer (PMO) that targeted the first 20 nucleotides of the 5′ UTR (located at the base of stem-loop A; Fig. 1). Resistance to the PMO evolved quickly (within two passages) as a result of two or three variants in the PMO binding sequence (103). A total of five mutations (G9U, G19A, C8G, C11G, and A23G) were identified in the resistant WNV 5′ UTRs, two of which were shared across at least two of the three replicates (G9U and G19A) (103). We identified G9U three times in our data set (robin passage 1 replicate A and sparrow passage 3 replicates A and B) at low frequencies (<2.3%). Additionally, we found that G9U occurs in a DS region and causes minor structure change in SLA. These results indicate that simply targeting oligomers to structures that are conserved across hosts will not prevent the evolution of resistance within hosts; instead, regions that are subject to purifying selection within hosts must be identified. To this end, the CYC binding site and UFS element may be ideal targets.

In sum, our study suggests that the intrahost population of WNV comprises a high degree of variation not just in primary RNA sequence but in RNA structure as well. Moreover, we detected no evidence of purifying selection acting to sieve out mutations that mediate structural change, even major structural changes, within hosts, save in the UFS stem structure. In their recent review of quasispecies theory, Domingo and Perales (5) expanded the mechanisms of quasispecies variation beyond point mutations to also include recombination, reassortment, gene duplication, and gene transfers. Our study suggests that, when considering the results of such mutations, variations in secondary structures should be considered in tandem with variations in the primary sequence.

## MATERIALS AND METHODS

### Serial passage of WNV and whole-genome sequencing.

As previously described in detail by Grubaugh et al. (64) WNVic (infectious clone 3356) (104) was injected into wild-caught American crows (Corvus brachyrhynchos), house sparrows (Passer domesticus), and American robins (Turdus migratorius). Three days postinfection, serum samples were collected and used to infect a new set of naive birds for a total of 5 passages, resulting in 45 virus populations sampled (five passages in three bird species each replicated in triplicate). Viral RNA was isolated and sequenced on the Illumina HiSeq 2000 platform (Beckman Coulter Genomics, Danvers, MA), and the data are available at the NCBI under BioProject number PRJNA281547 (64).

### Identification of variants.

Part of SLB and all of cHP, as well as three of the binding sites (Fig. 1a), extend from the 5′ UTR into the capsid gene (62). Thus, for this analysis, we included the whole 5′ UTR and the first 54 nucleotides of the ORF to incorporate all of these elements, for a total region of 150 nucleotides. We compiled the variants that occurred at a frequency of ≥1.0% for passages 1, 3, and 5 for all three bird hosts. All variant frequencies were calculated using Geneious version 9.0.5 (105).

### Prediction of secondary structure and classification of structure changes.

The secondary structure of the first 150 nt of wild-type WNVic (GenBank accession no. AF404756.1) was predicted using the online mFold application version 4.7 (http://unafold.rna.albany.edu/ [74]). The default folding parameters were utilized for a linear RNA sequence, which includes a fixed folding temperature of 37°C, 1 M NaCl, 5% suboptimality, maximum interior loop size of 30 nt, and maximum interior loop asymmetry of 30 nt. The resulting structure was identical to previously documented secondary structures for the WNV 5′ UTR (55, 62).

To determine the structure of variant haplotypes, each specific nucleotide variation from the wild-type sequence was identified, and the corresponding change was made to a template sequence comprising the 150 nt at the 5′ end of the wild-type WNV genome (Fig. 1a). Each variant sequence was folded using mFold, as described above. Two properties of each nucleotide variant were noted, as follows: (i) whether the nucleotide occurred in a DS or SS region of the wild-type structure, and (ii) whether the variant occurred in one of four sites known to bind sequences in the 3′ UTR, namely, UAR (upstream of start codon), DAR I, DAR II (downstream of start codon), and CYC (cyclization sequence) (Fig. 1a) (62). Variants of the haplotypes that contained two or three mutations were folded individually and in combination. Unless otherwise noted, only the RNA structures resulting from individual variants were used for analyses. To test the sensitivity of our results to our choice of folding program, we assigned a numerical code to each of the unique structures and used a random number generator to create a subset of 20% of total structures, which we refolded in RNAstructure version 6.0.1 at 37°C. Additionally, these sequences were also folded using RNAstructure version 6.0.1 at 40°C, the average bird body temperature (106). RNAstructure was used to test the effect of folding temperature because at 40°C, mFold version 2.3 does not recapitulate the wild-type WNV 5′ UTR structure (62). As crows experience a high fever during WNV infection (107), the crow viral variants from passage 5 were also folded using RNAstructure at 42°C.

Based on the folded structure, each variant was classified as causing a major structure change, a minor structure change, or no structure change (i.e., the structure was identical to the wild type). A major structure change was defined as six or more changes in strandedness, i.e., SS to DS or DS to SS, while a minor structure change was fewer than six but more than zero changes in strandedness.

### Statistical analysis.

The distribution of variants causing structure change (major or minor) and variant location (DS or SS) was analyzed using contingency analyses to obtain a χ^2^ statistic or Fisher’s exact *P* value, with Bonferroni’s correction where applicable. The Kruskal-Wallis rank sum test was used to detect correlations between host-specific viral fitness and structure change. As described by Assis (45), the proportion of mutations in DS or SS sites (number of mutations in DS or SS sites divided by total number of DS or SS nucleotides) and the proportion of DS or SS sites that are saturated with mutations (calculated as the [number of mutations in DS or SS sites divided by the total number of DS or SS nucleotides multiplied by 3] × 100) were calculated for each bird and passage. An exact binomial test was then used to detect differences in mutation saturations in the DS and SS sites. Exact binomial tests were also conducted for each binding site and the UFS sequence. Statistics were conducted using R (https://www.r-project.org/ [108]).

### Data availability.

The sequencing data utilized for this project have been deposited in the NCBI Sequence Read Archive database and can be accessed with the BioProject identifier PRJNA281547.
