# Supplementary material for: Endless Forms: Within-Host Variation in the Structure of the West Nile Virus RNA Genome during Serial Passage in Bird Hosts
Source: mSphere. 2019 Jun 26;4(3):e00291-19. doi: 10.1128/mSphere.00291-19 (PMC6595145; doi:10.1128/mSphere.00291-19)
Supplement: TABLE S1 [file mSphere.00291-19-st001.docx]

| 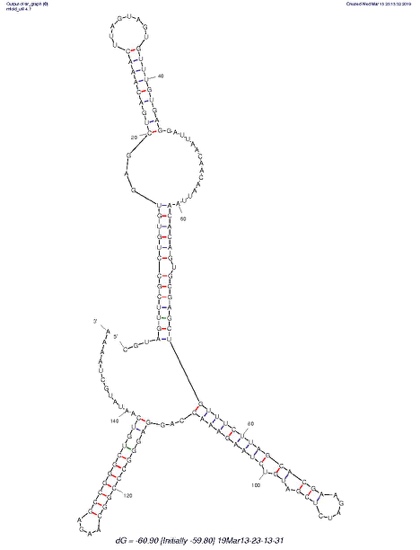  A1C | 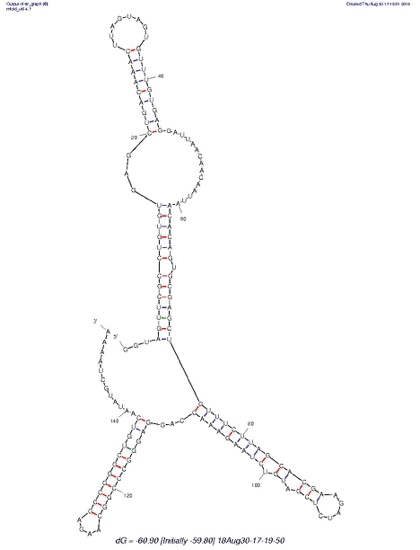  A1G | 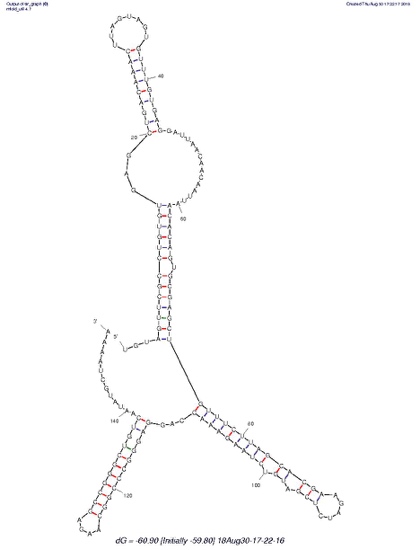  A1T |
| --- | --- | --- |
| 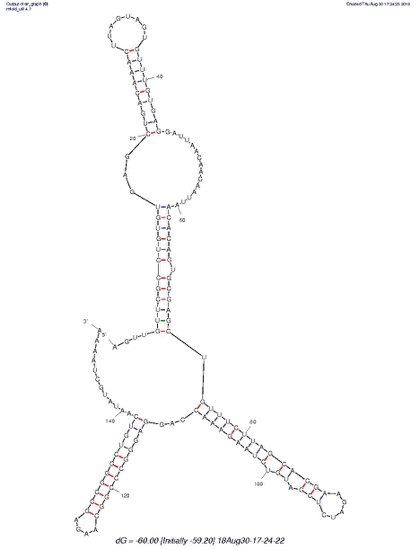  A4T | 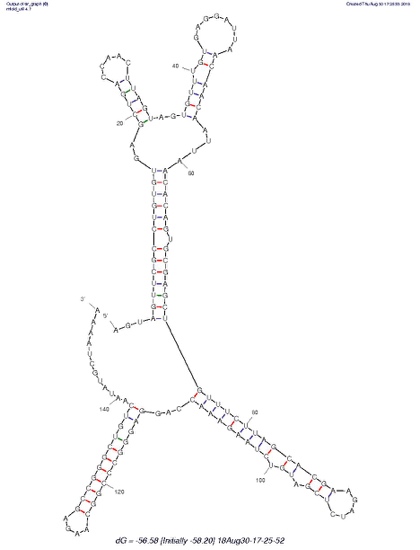  A25C | 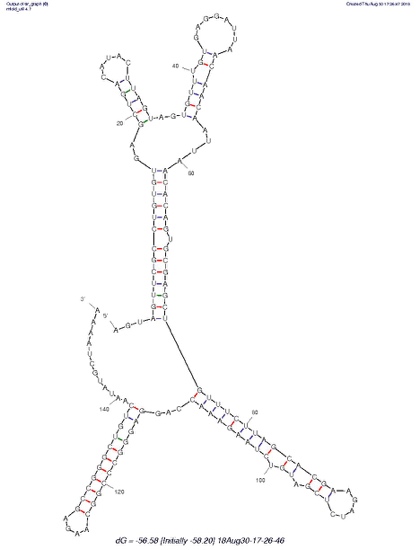  A26T |
| 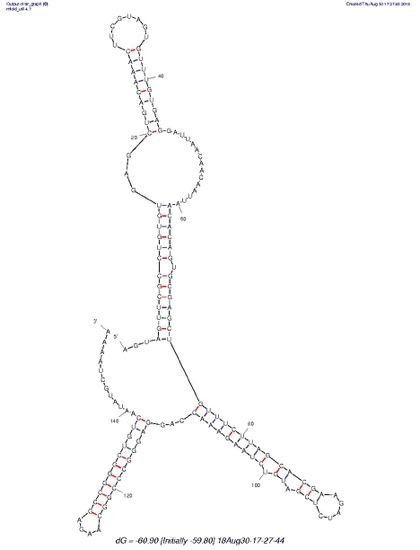  A31C | 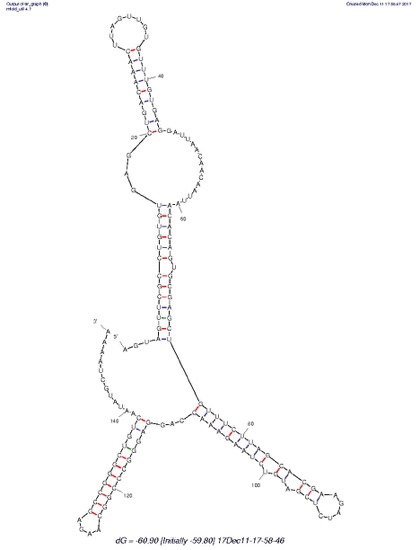  A34T | 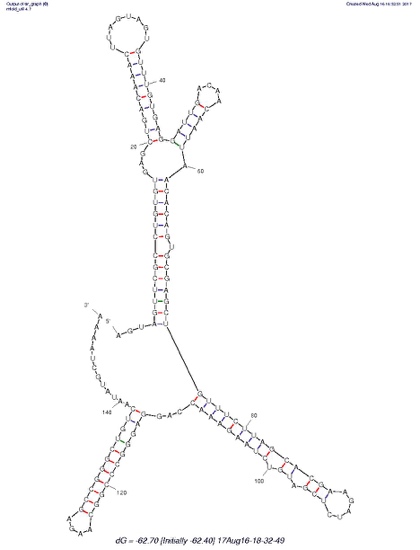  A50G |
| 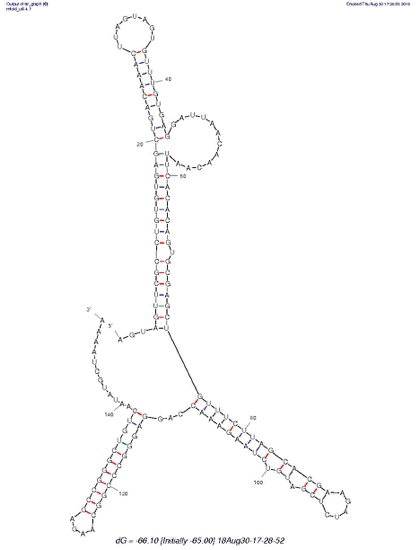  A60C | 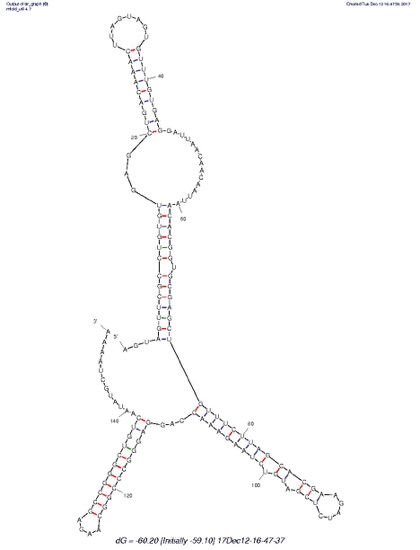  A65G | 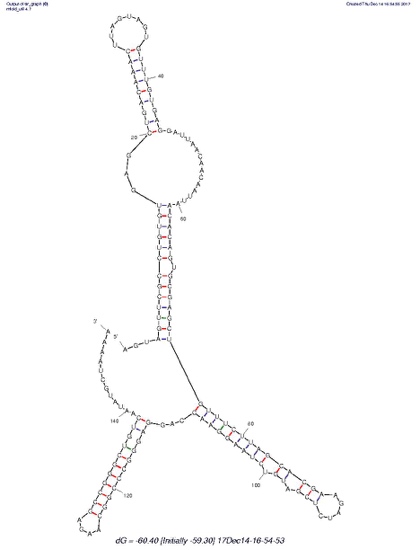  A106G |
| 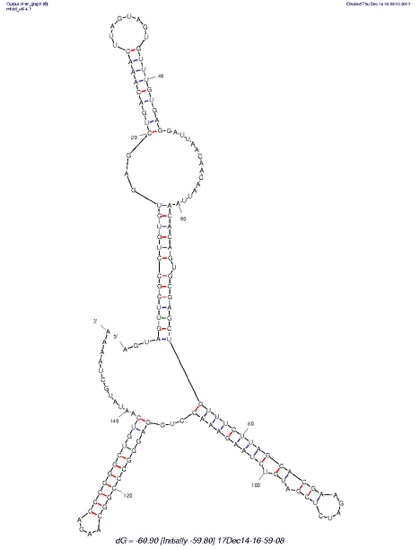  A111T | 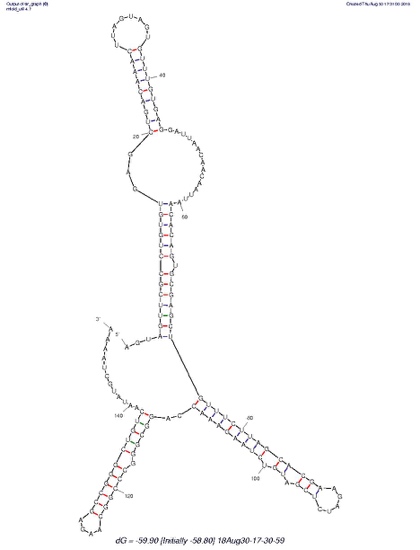  A114C | 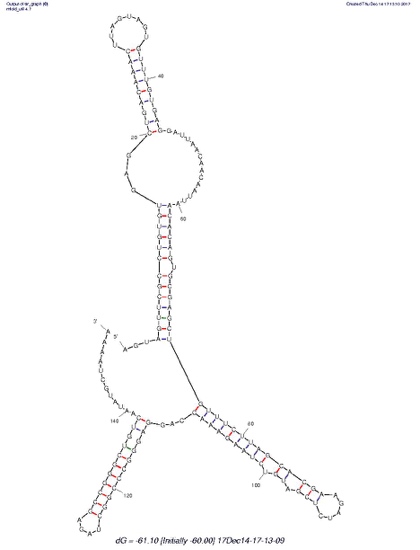  A124T |
| 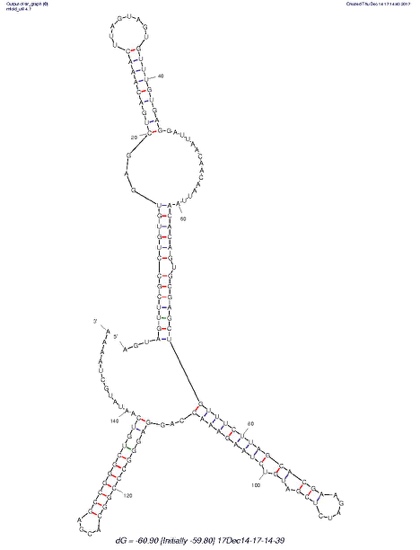  A125C | 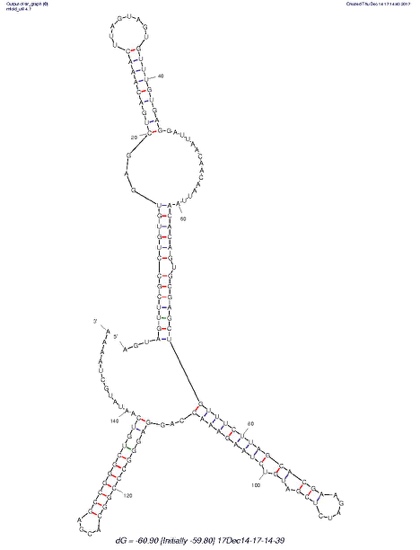  A125C | 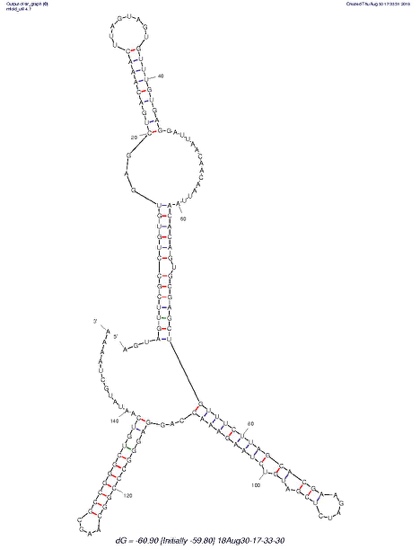  A127C |
| 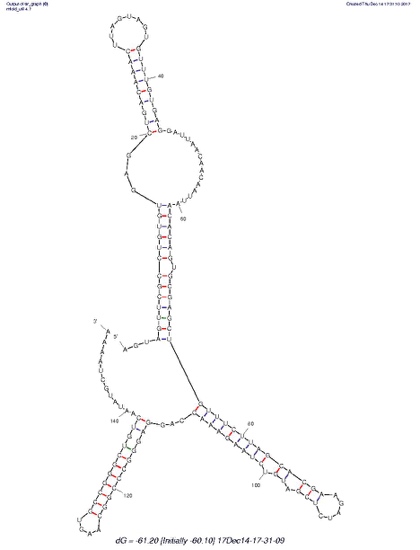  A127T | 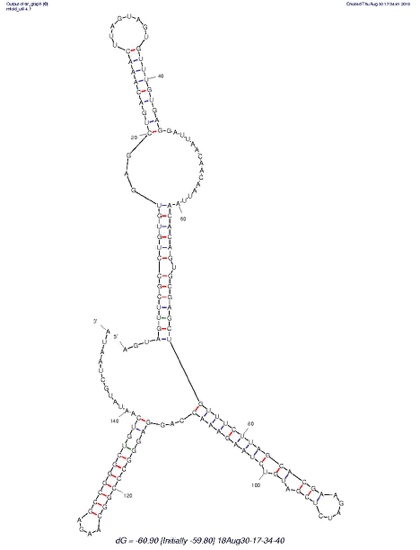  A149T | 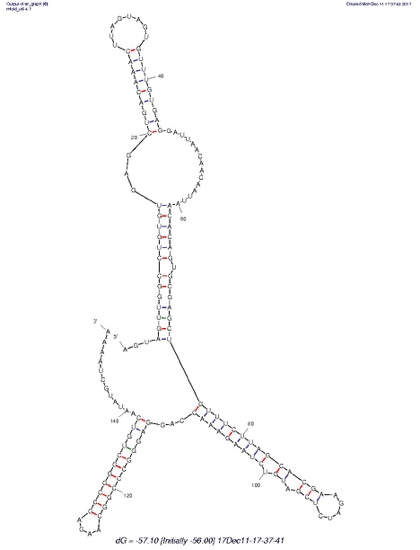  C8G |
| 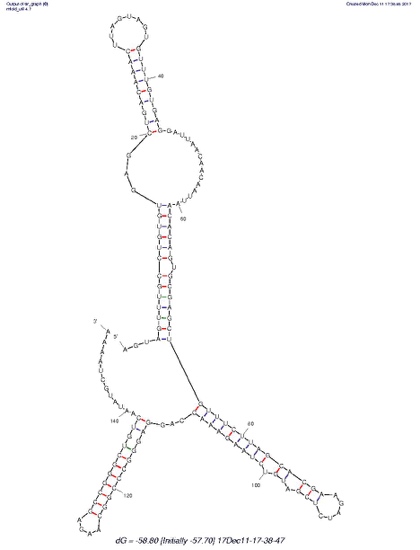  C8T | 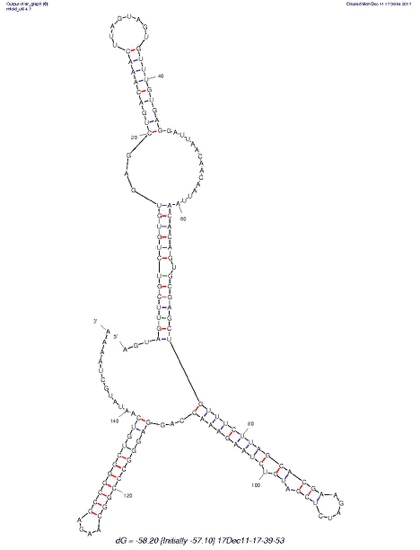  C10T | 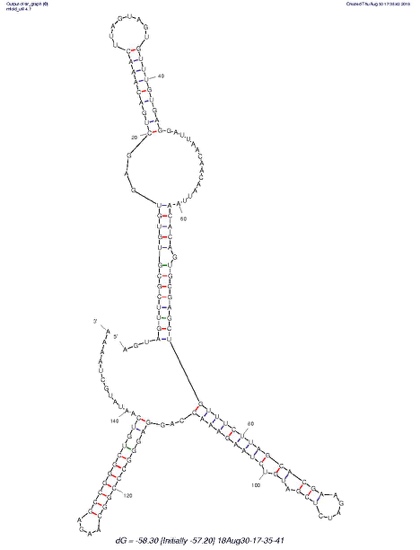  C11G |
| 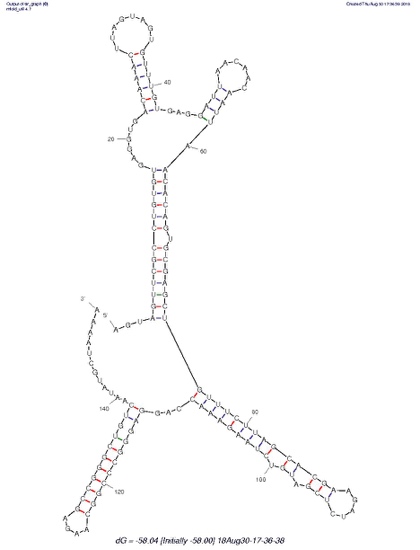  C20G | 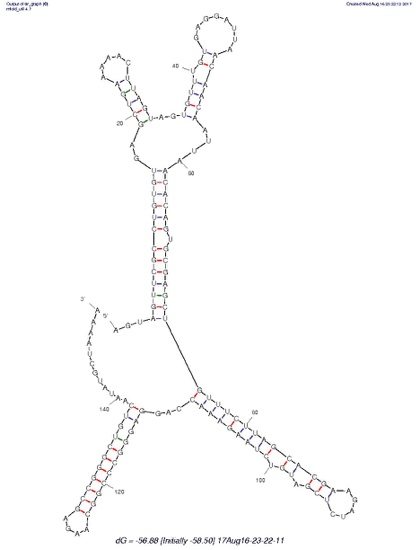  C24A | 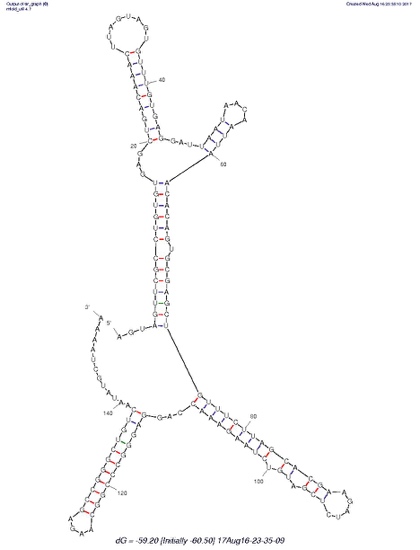  C52T |
| 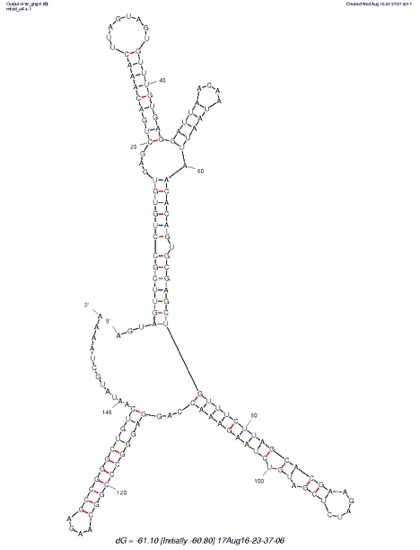  C55T | 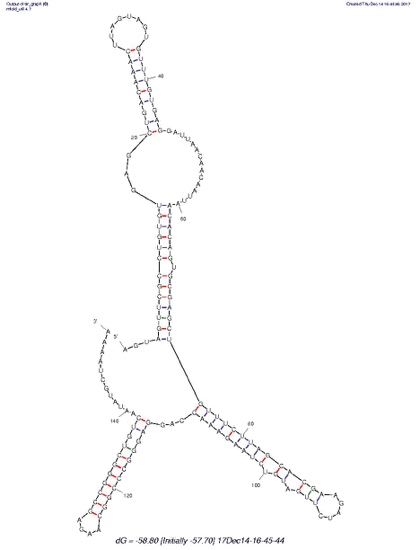  C95T | 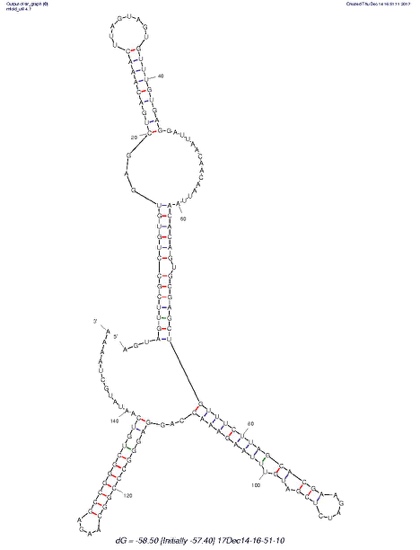  C101T |
| 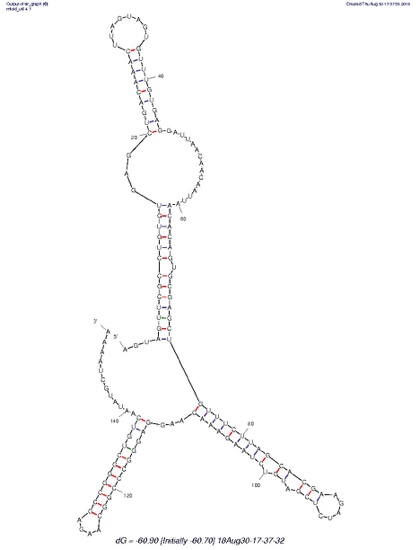  C110A | 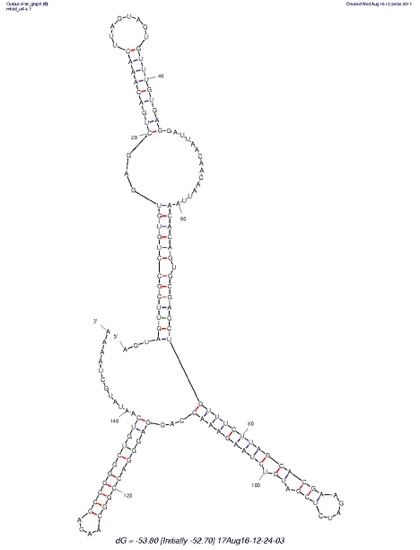  C118A | 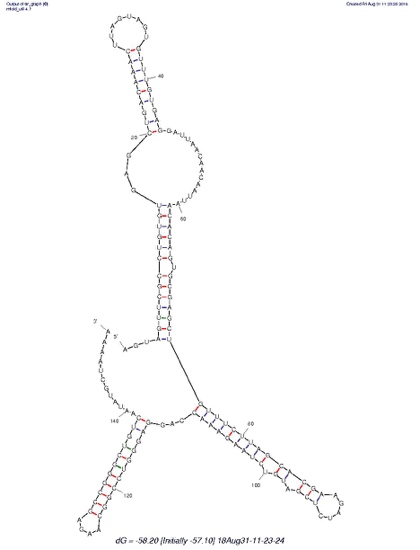  C118T |
| 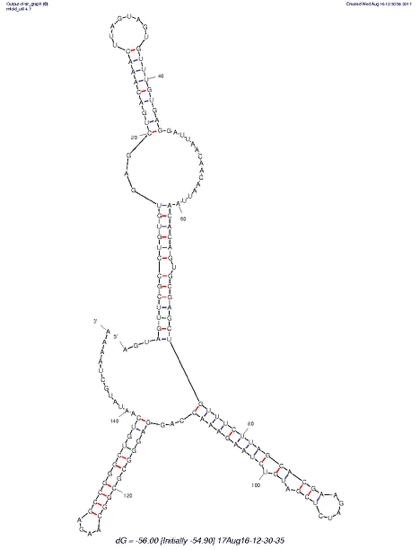  C119G | 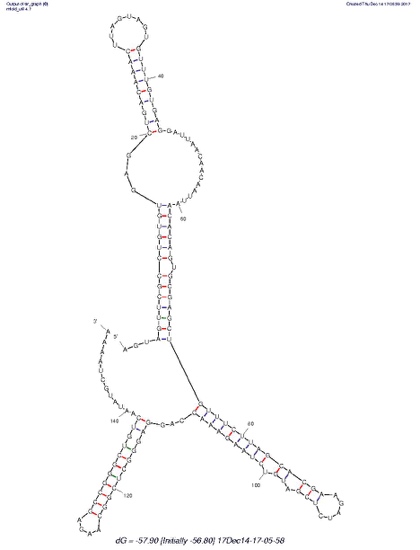  C119T | 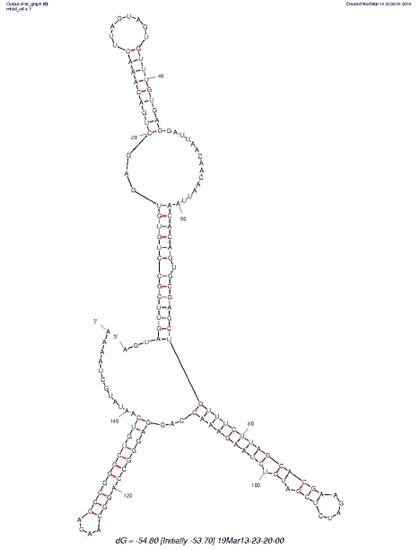  C120A |
| 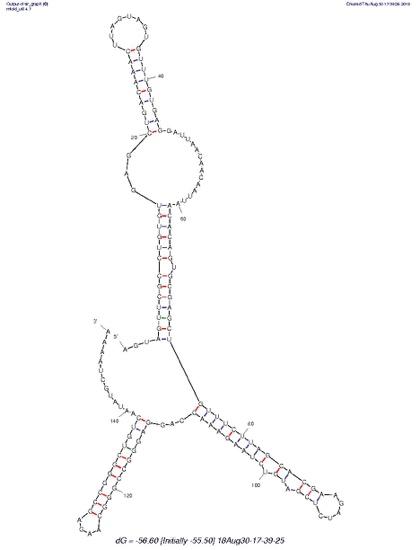  C120G | 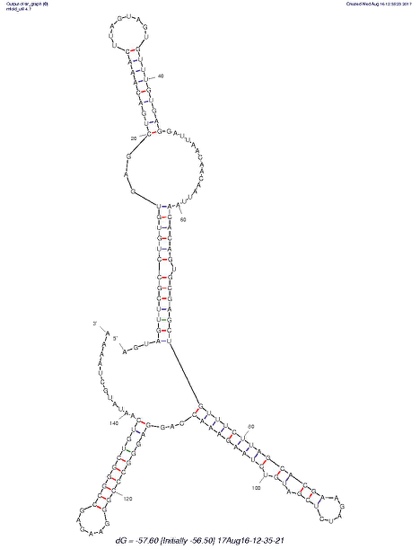  C123G | 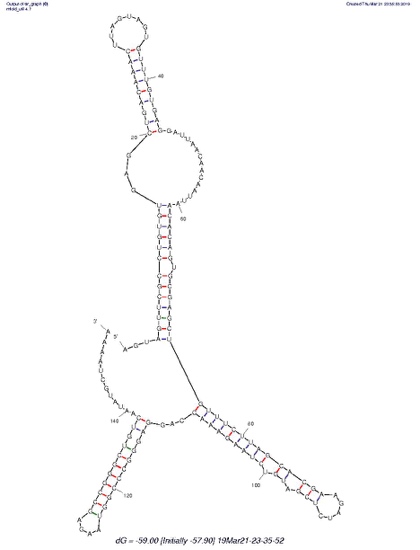  C123T |
| 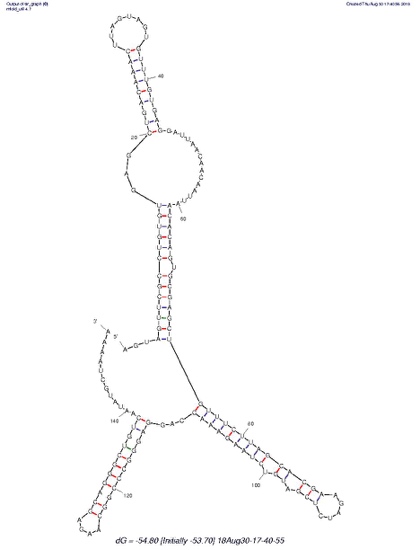  C130A | 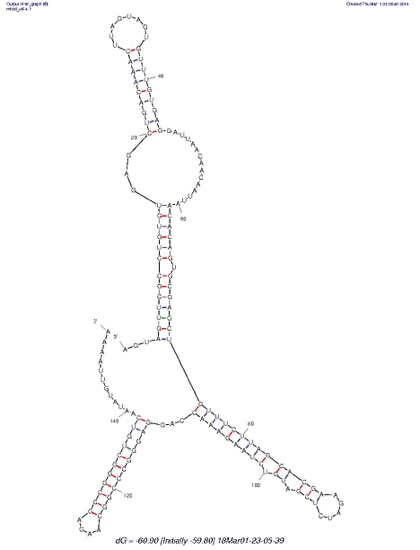  C145T | 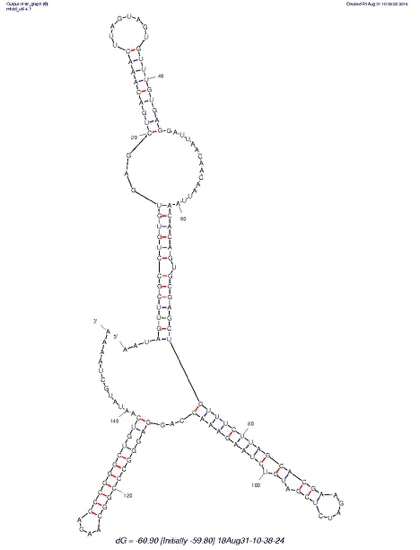  G2A |
| 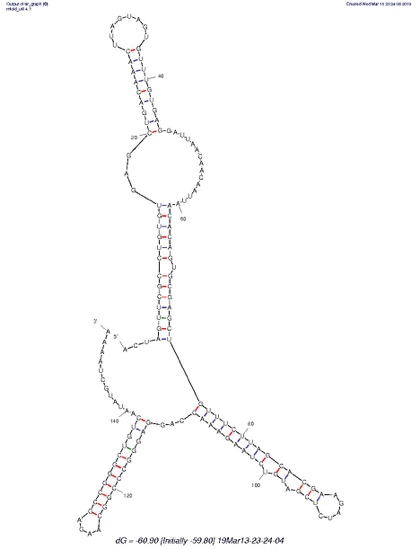  G2C | 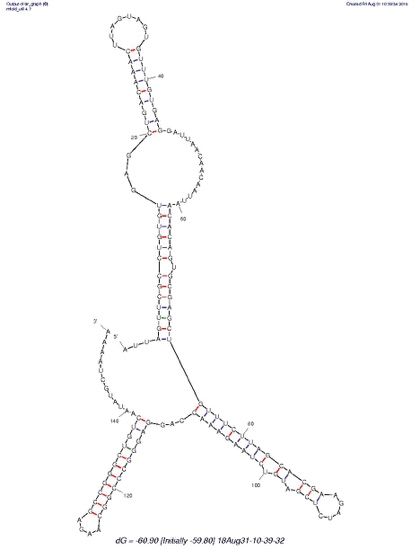  G2T | 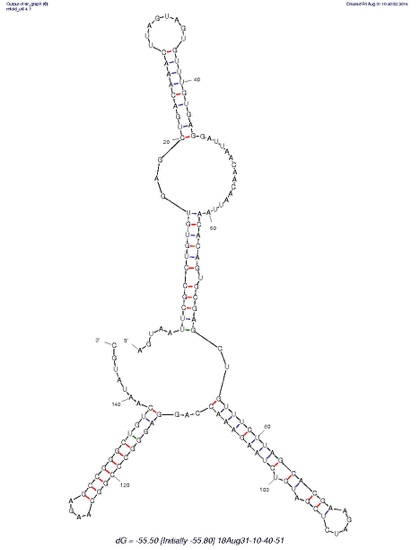  G5A |
| 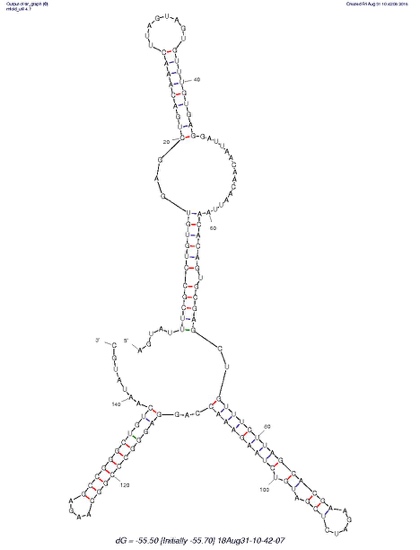  G5T | 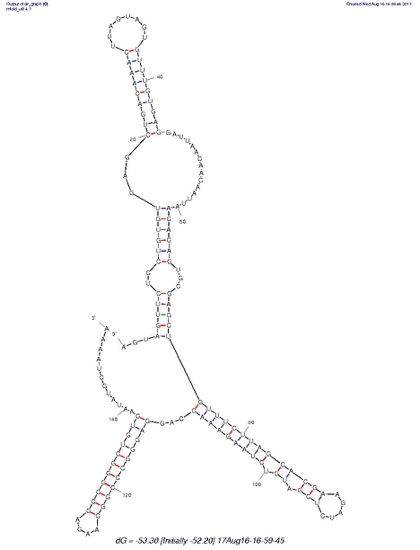  G9T | 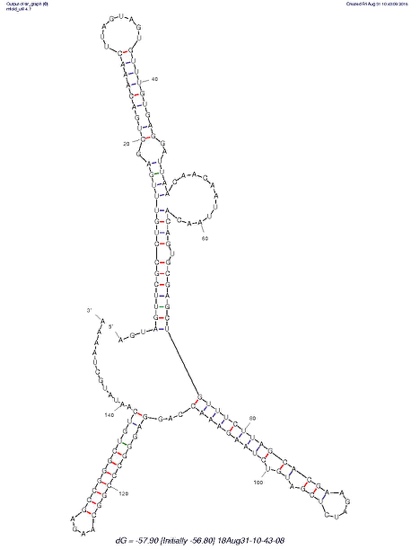  G15T |
| 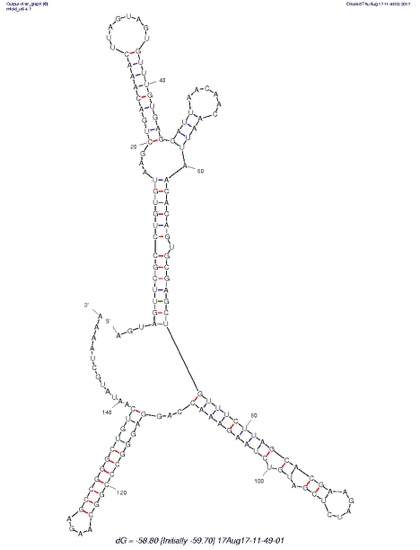  G17A | 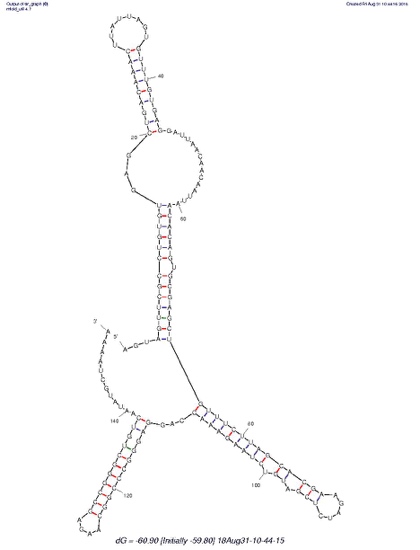  G32T | 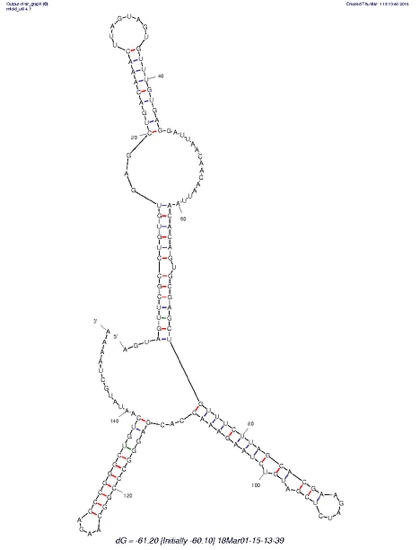  G112C |
| 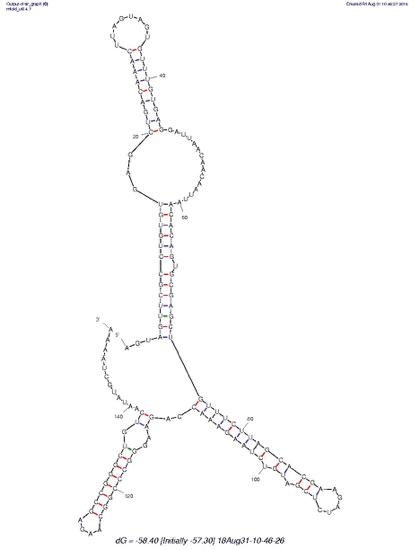  G113A | 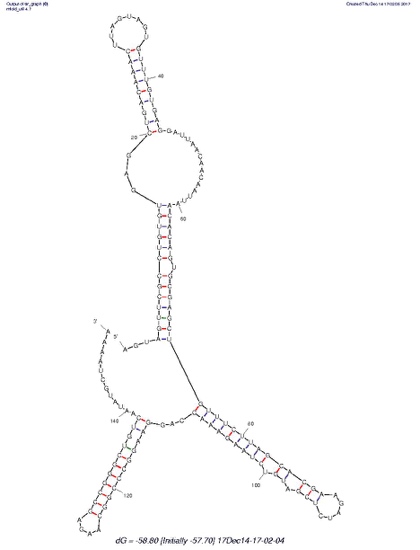  G115A | 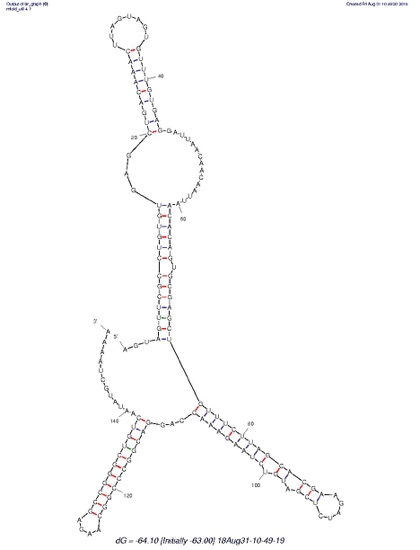  G115C |
| 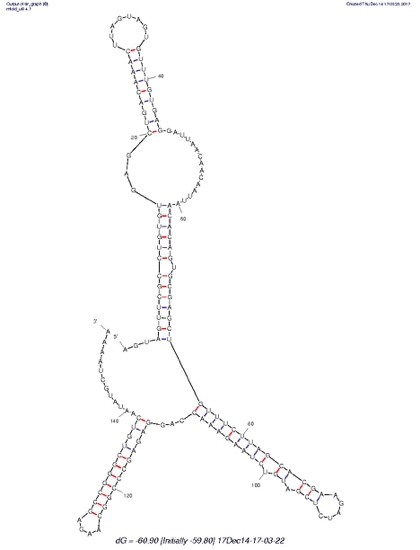  G116A | 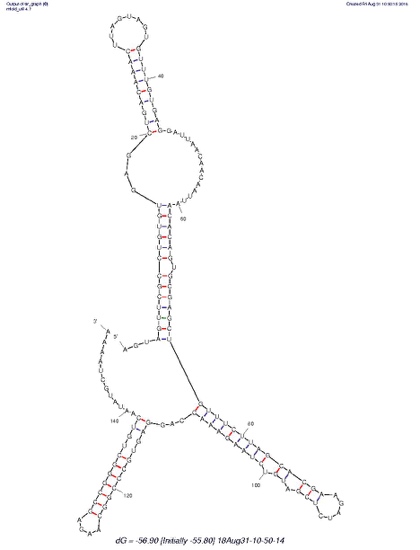  G116T | 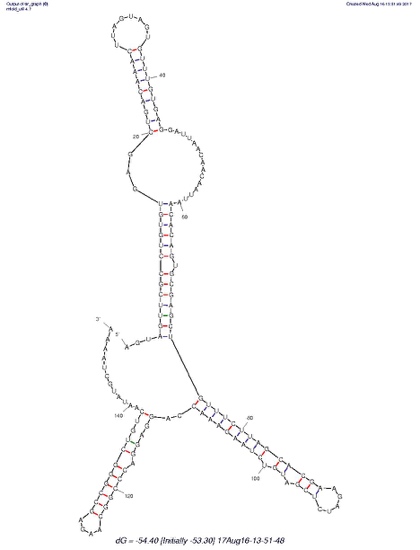  G117A |
| 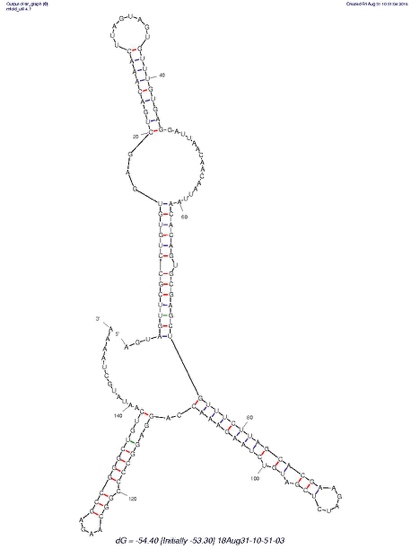  G117C | 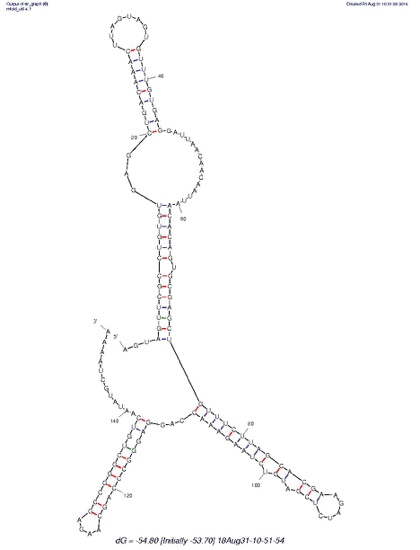  G121A | 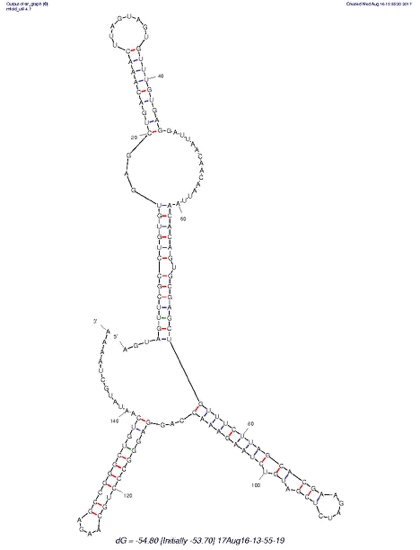  G121T |
| 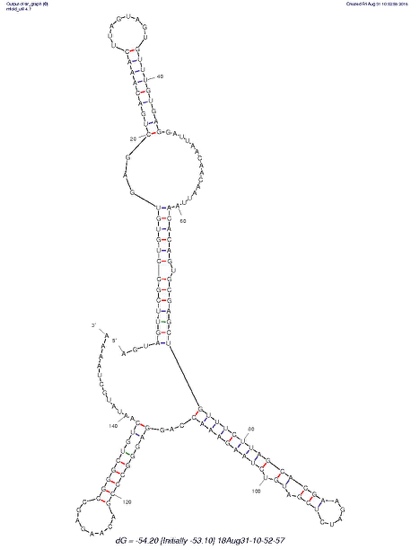  G122A | 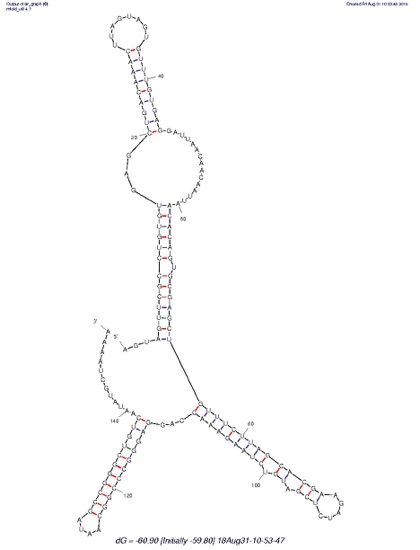  G126T | 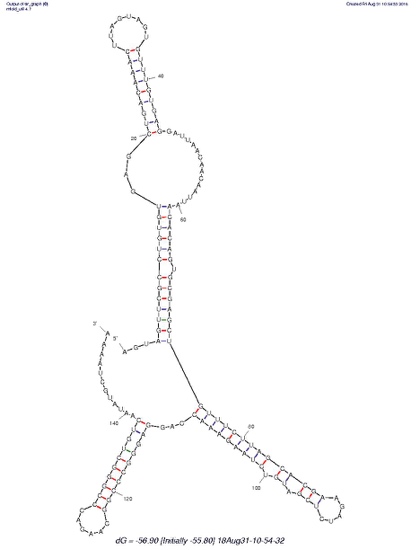  G128C |
| 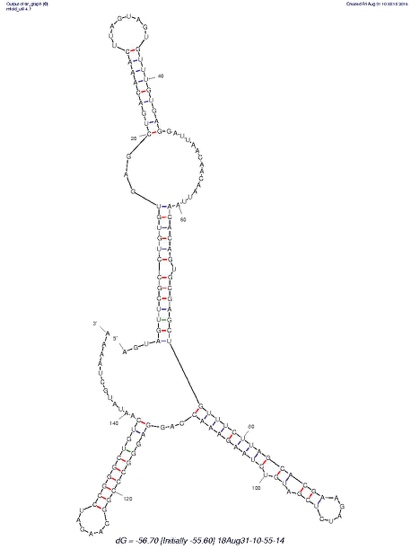  G128T | 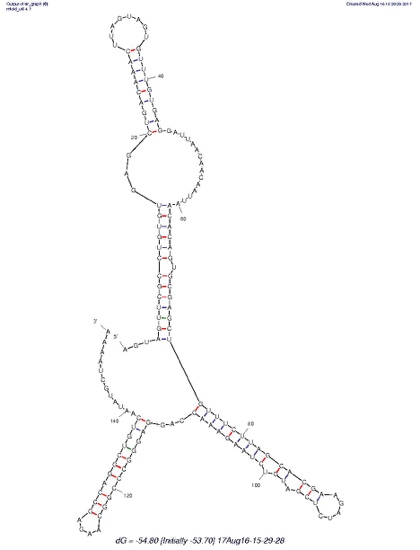  G131A | 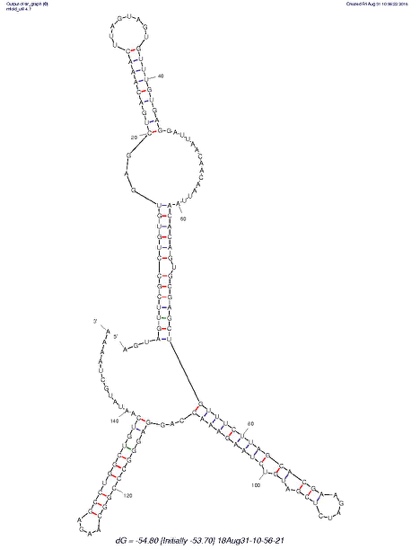  G131T |
| 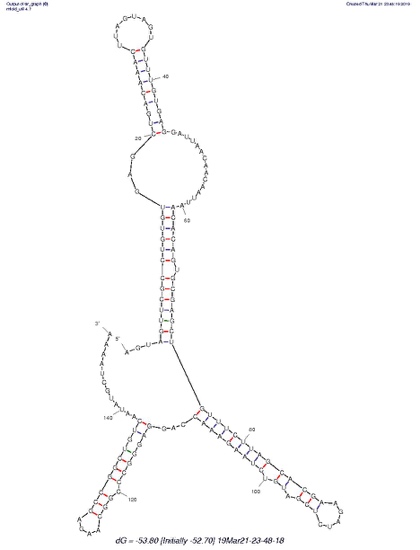  G132C | 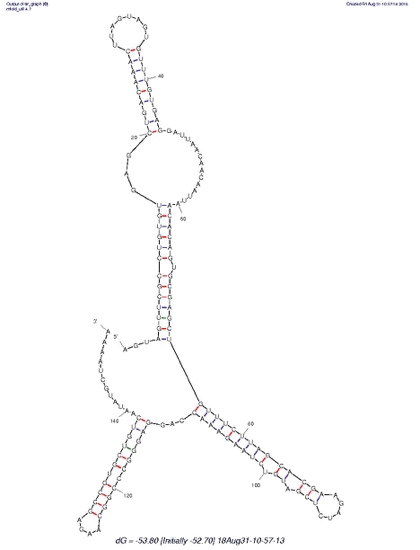  G132T | 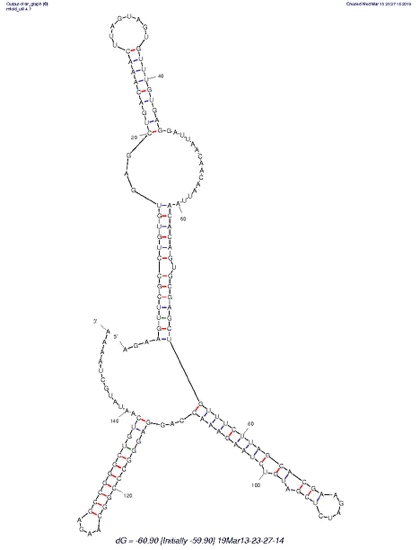  T3A |
| 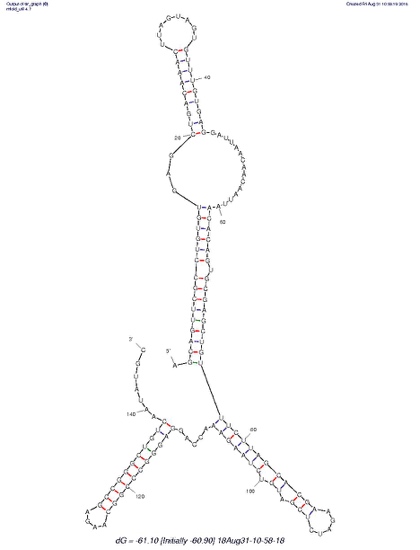  T3C | 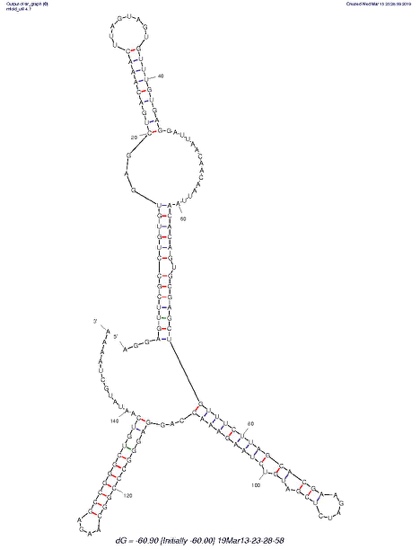  T3G | 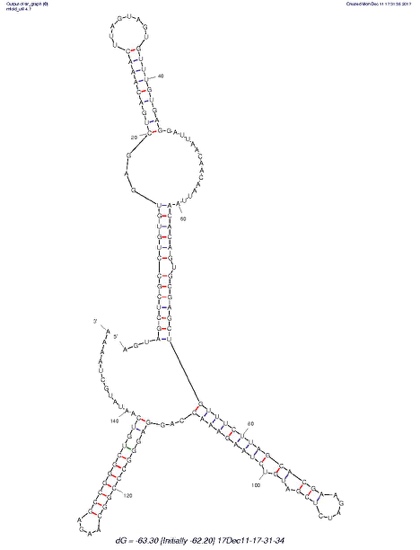  T6C |
| 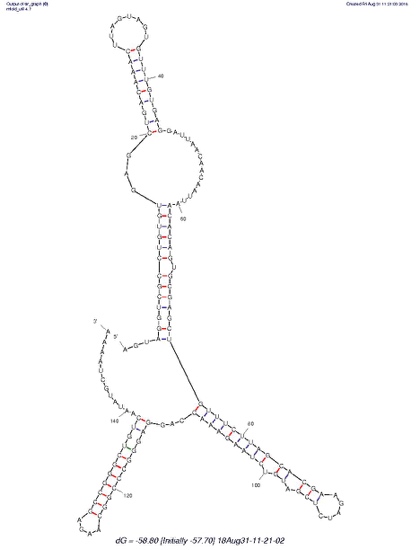  T6G | 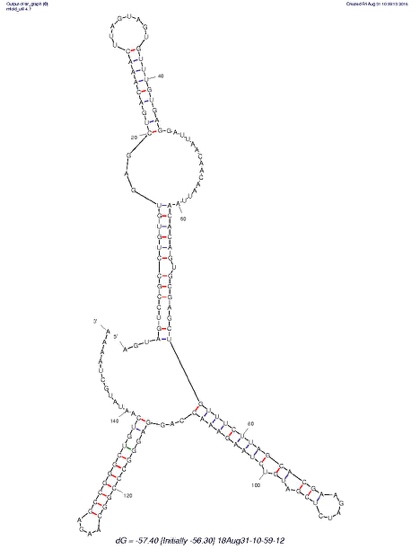  T7C | 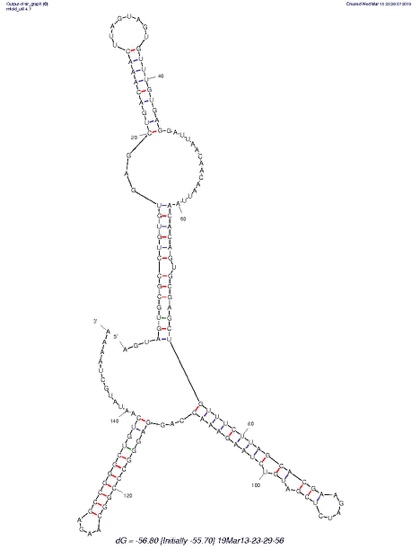  T7G |
| 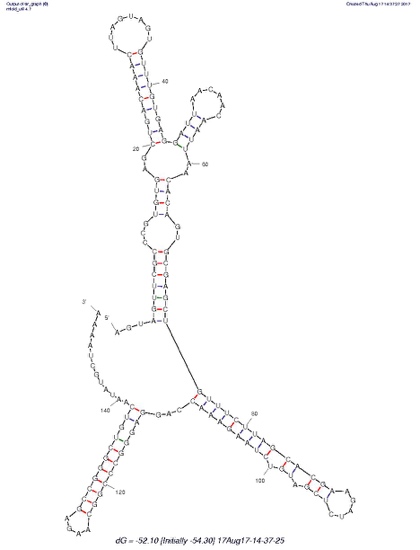  T12C | 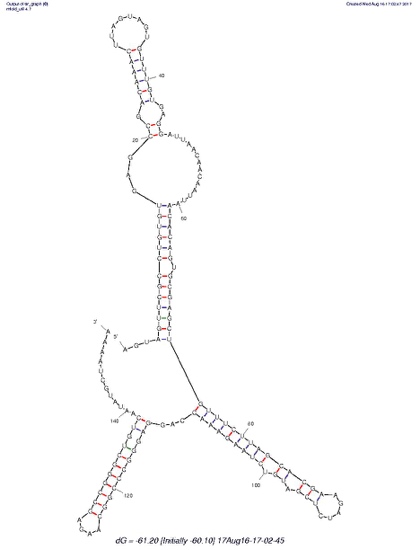  T21C | 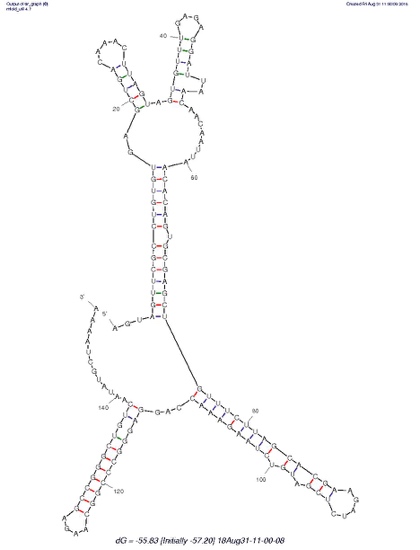  T42A |
| 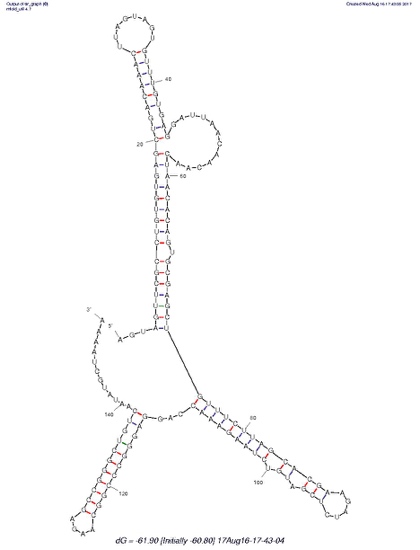  T58C | 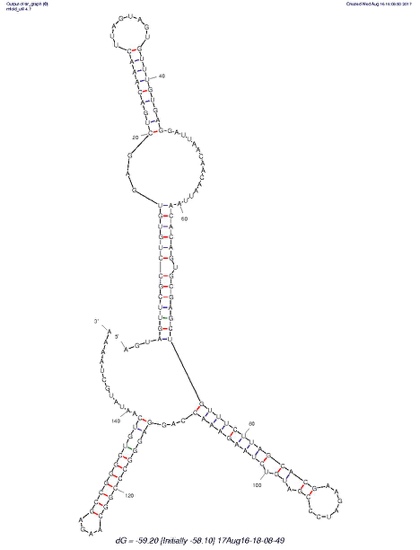  T94C | 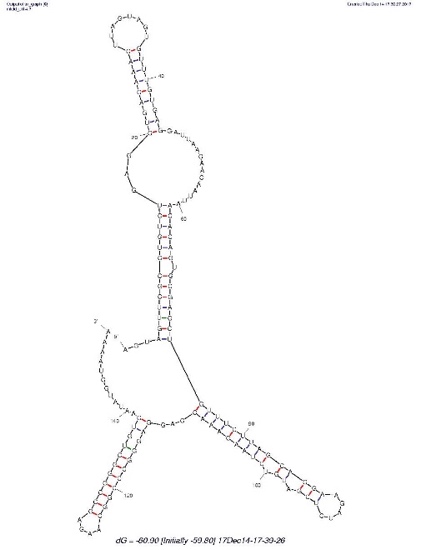  Wild-type |
|  |  |  |
