# Supplementary material for: Endless Forms: Within-Host Variation in the Structure of the West Nile Virus RNA Genome during Serial Passage in Bird Hosts
Source: mSphere. 2019 Jun 26;4(3):e00291-19. doi: 10.1128/mSphere.00291-19 (PMC6595145; doi:10.1128/mSphere.00291-19)
Supplement: TABLE S2 [file mSphere.00291-19-st002.docx]

|  | mFold at  37°C | RNAstructure at 37°C | RNAstructure  at 40°C |
| --- | --- | --- | --- |
| A26T | 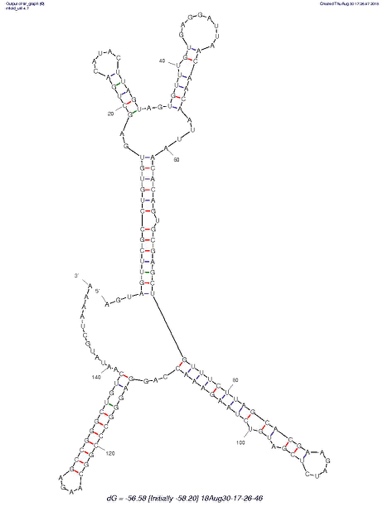  Major | 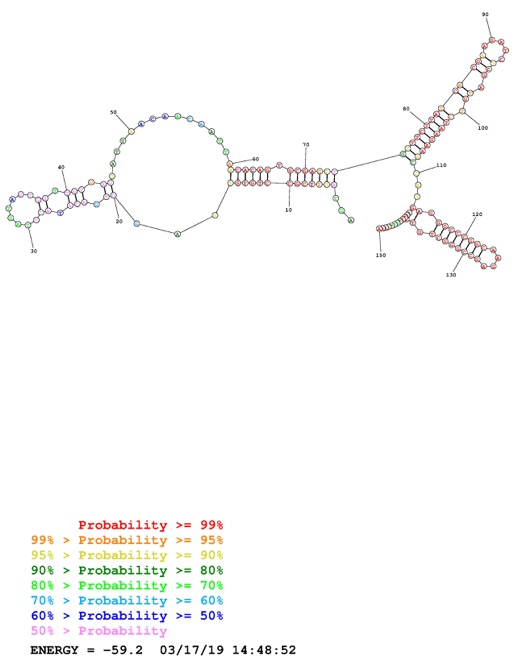  Minor | 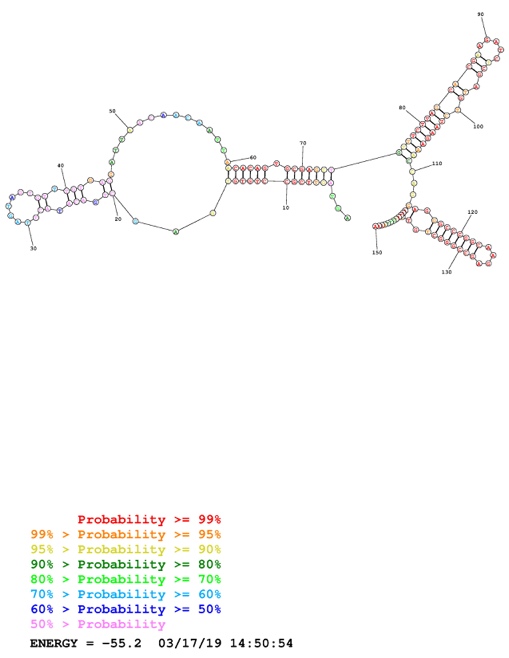  Minor |
| A34G | 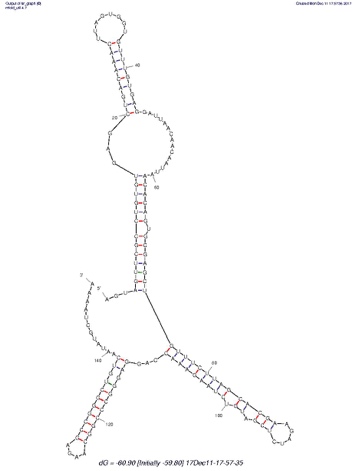  None | 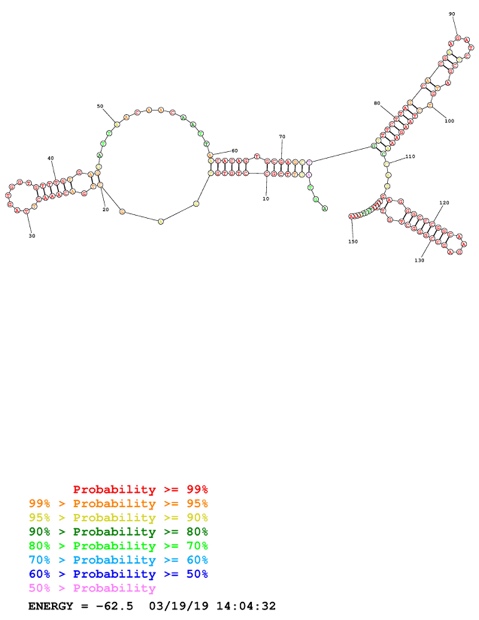  None | 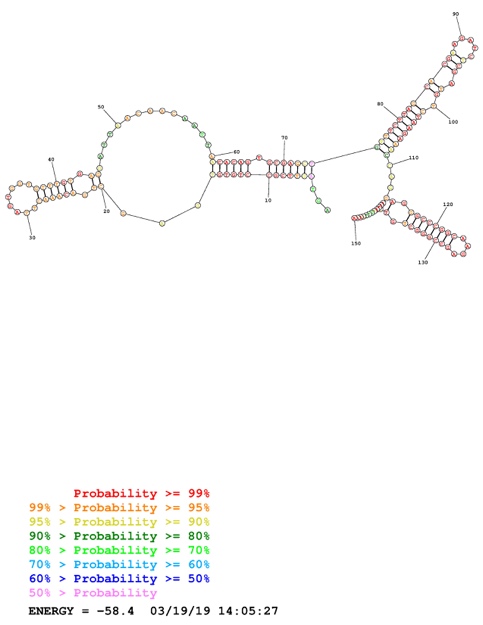  None |
| A50G | 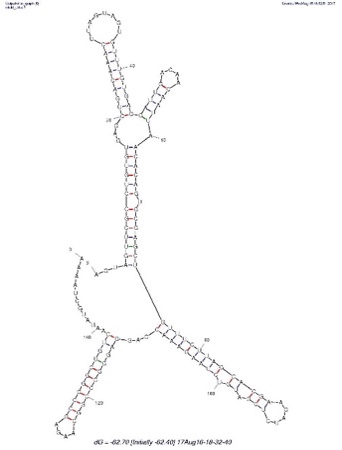  Major | 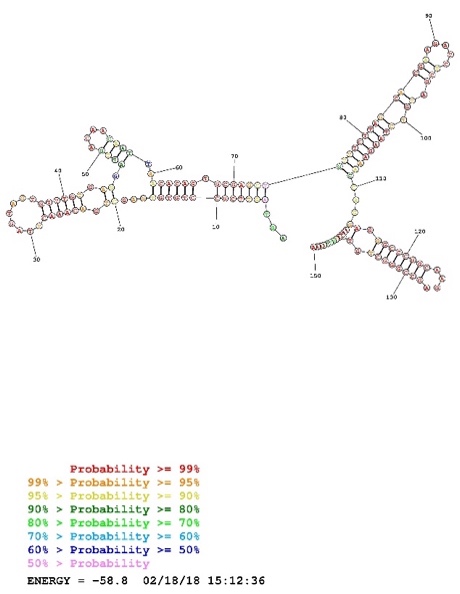  Major | 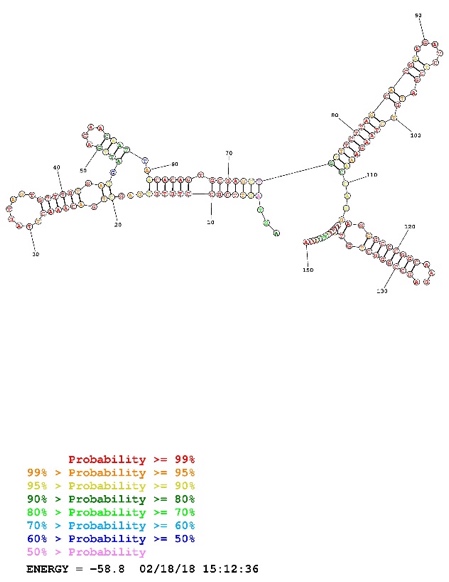  Major |
|  |  |  |  |
| A65G | 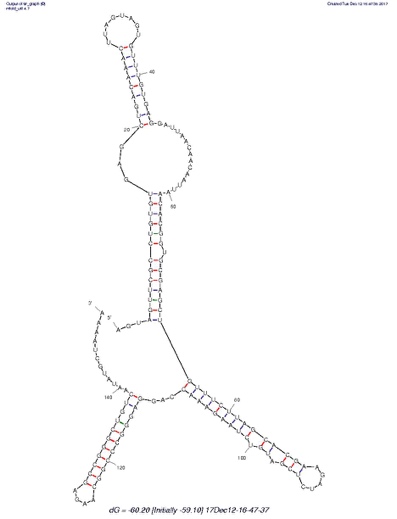  None | 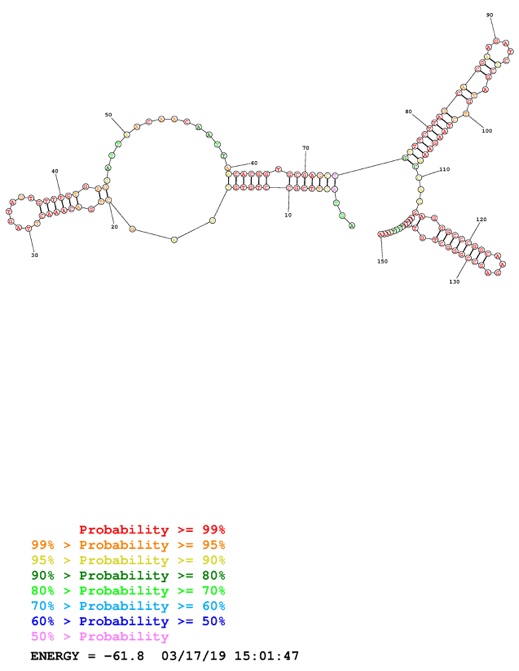  None | 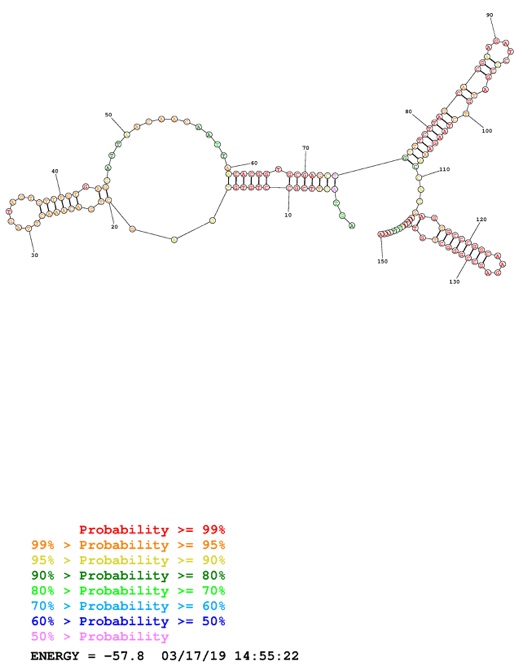  None |
| A106G | 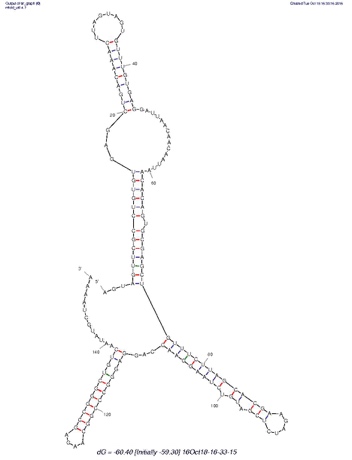  None | 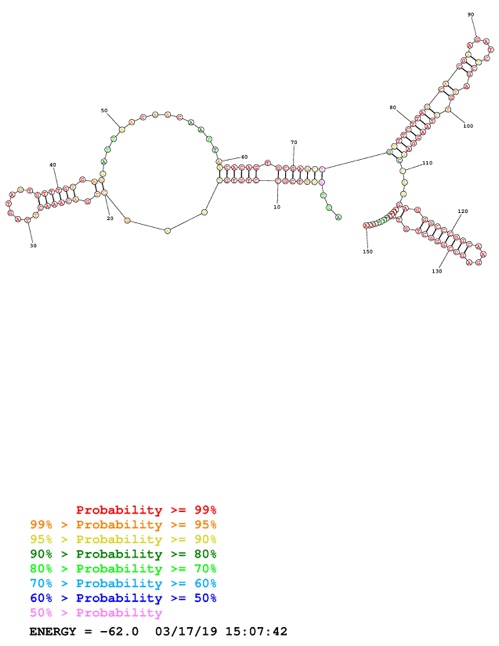  None | 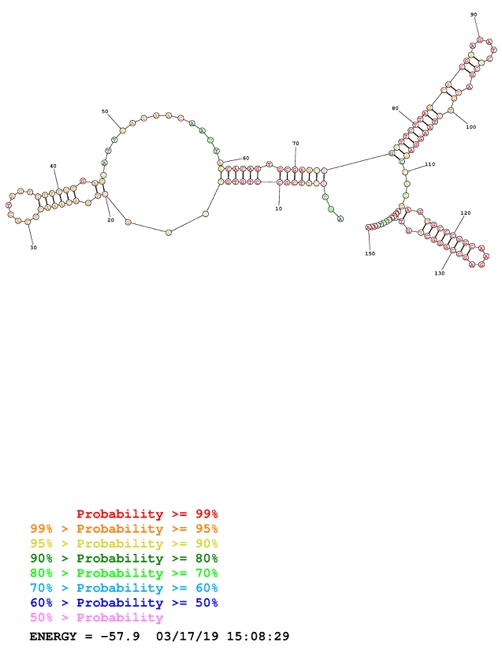  None |
| A111T | 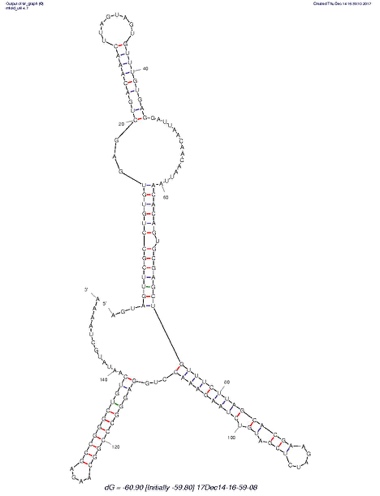  None | 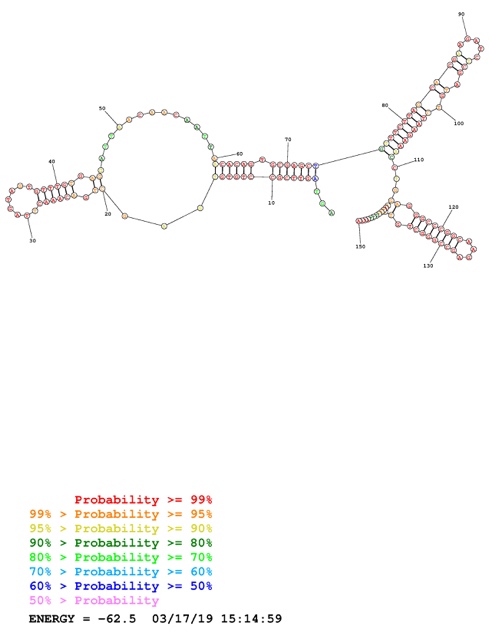  None | 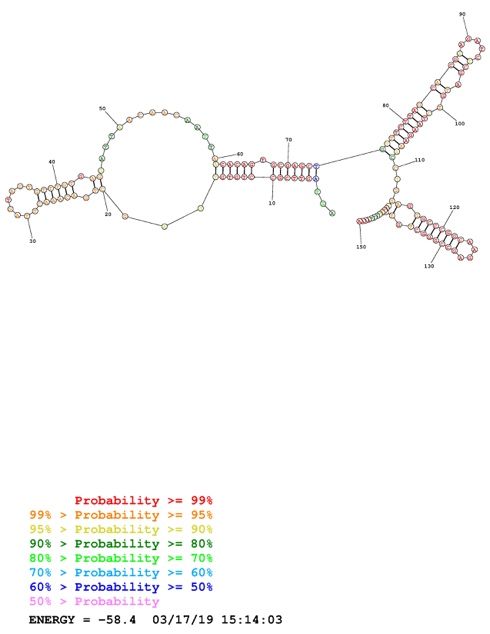  None |
| A127T | 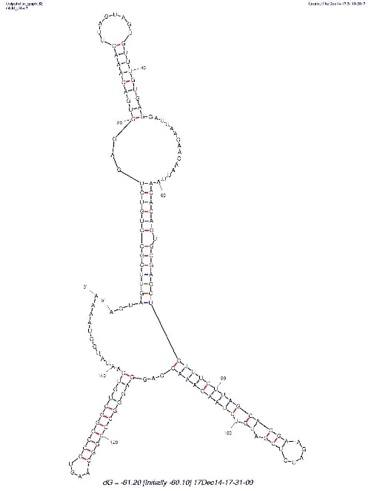  None | 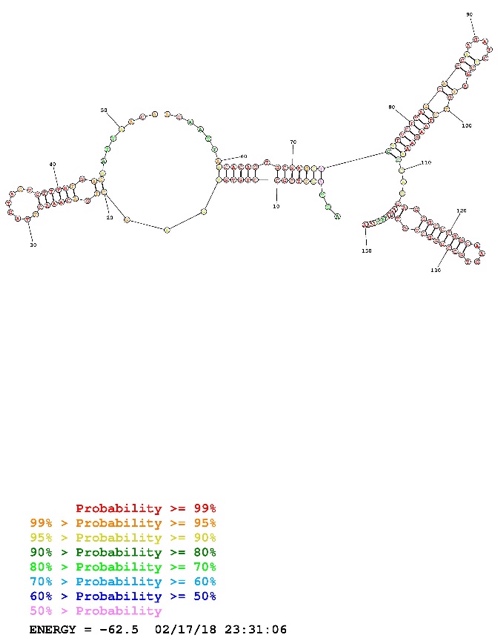  None | 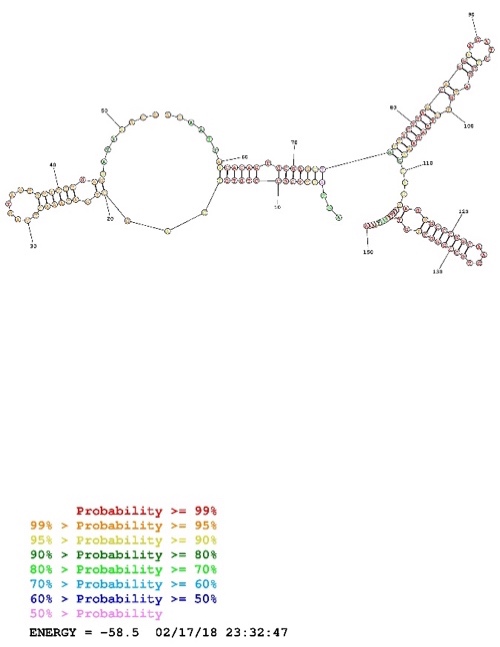  None |
| C10T | 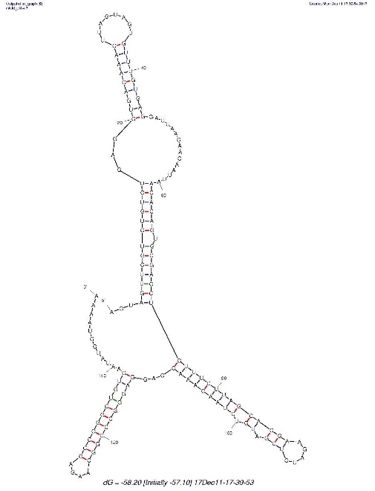  None | 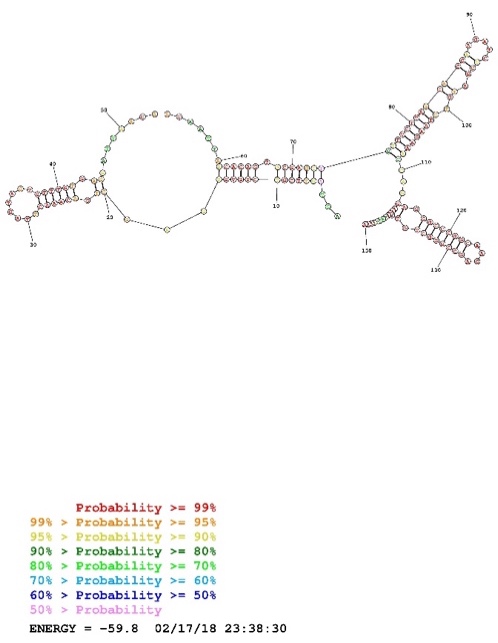  None | 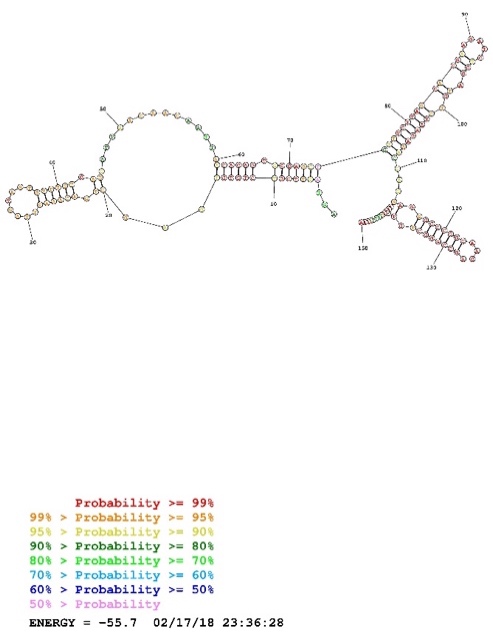  None |
| C118A | 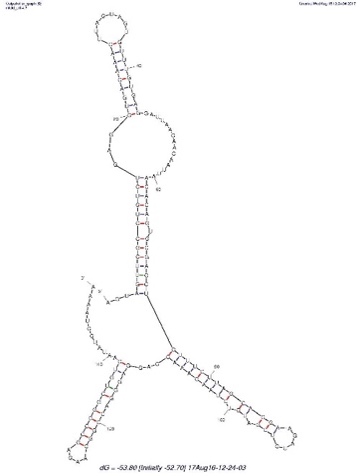  Minor | 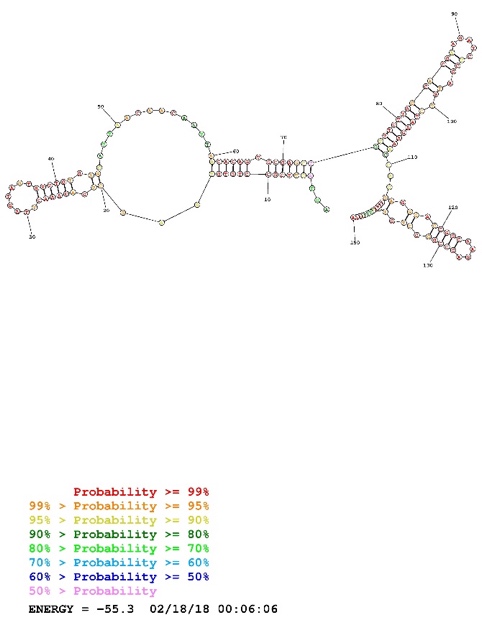  Minor | 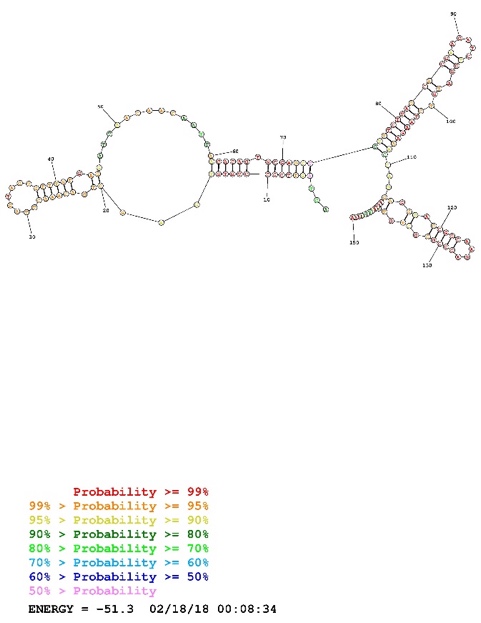  Minor |
| C119G | 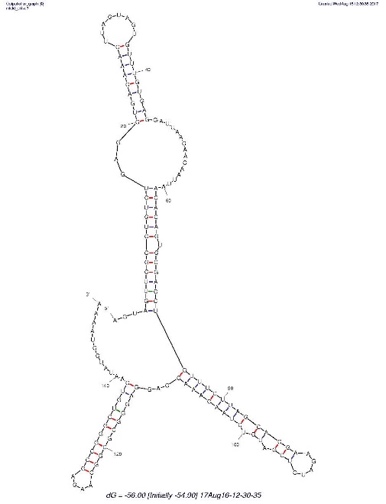  Minor | 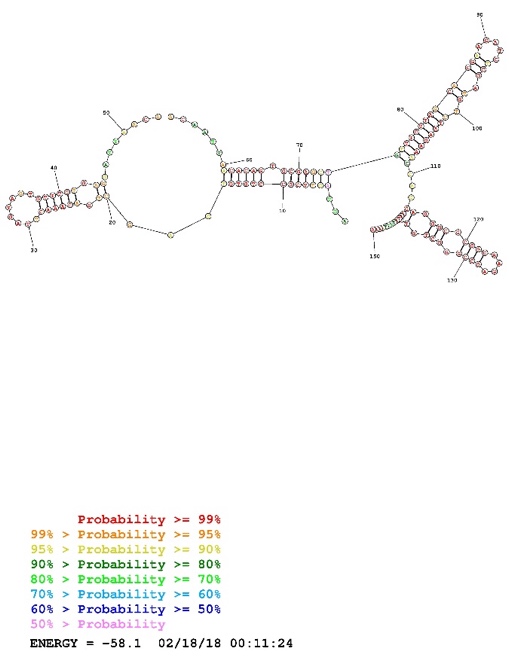  Minor | 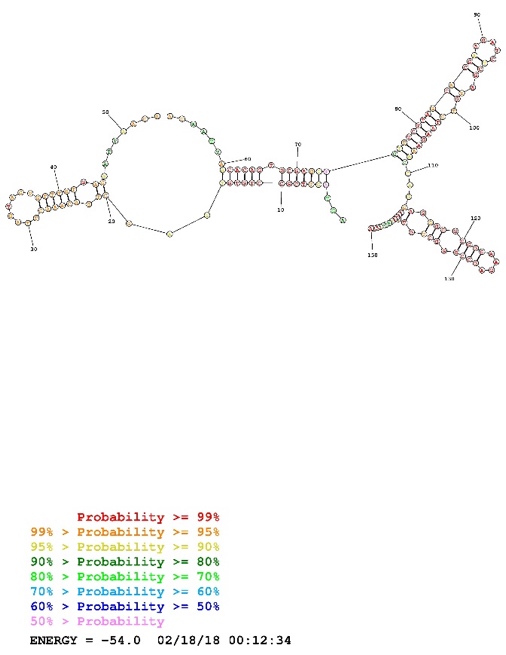  Minor |
| G115A | 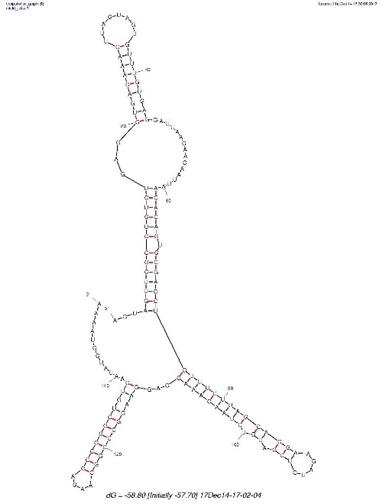  None | 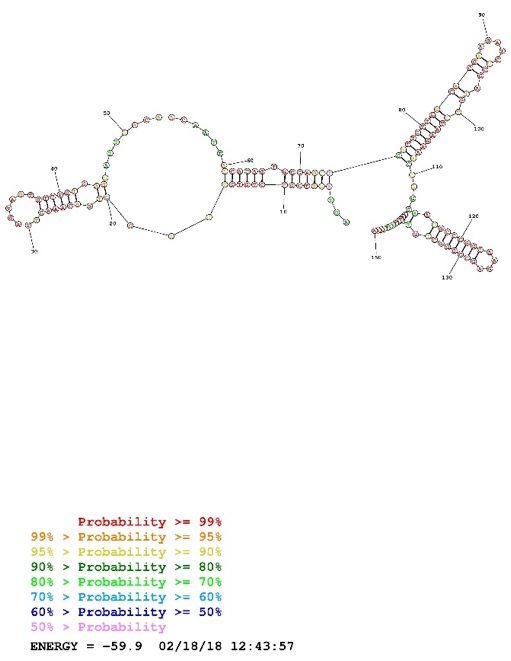  None | 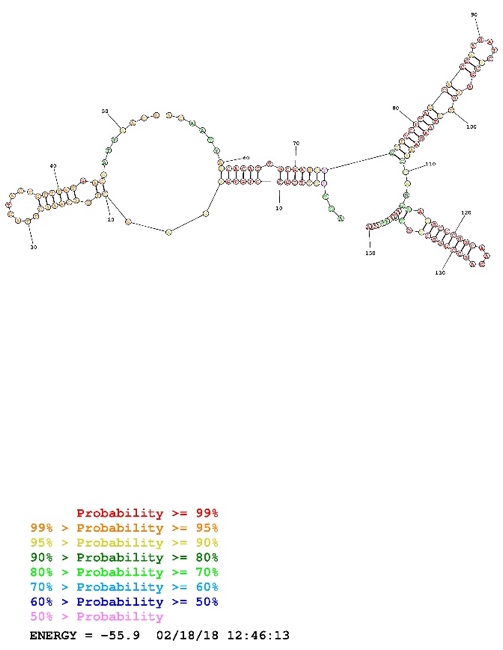  None |
| G117A | 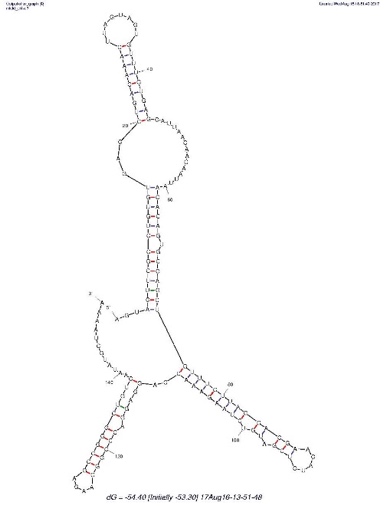  Major | 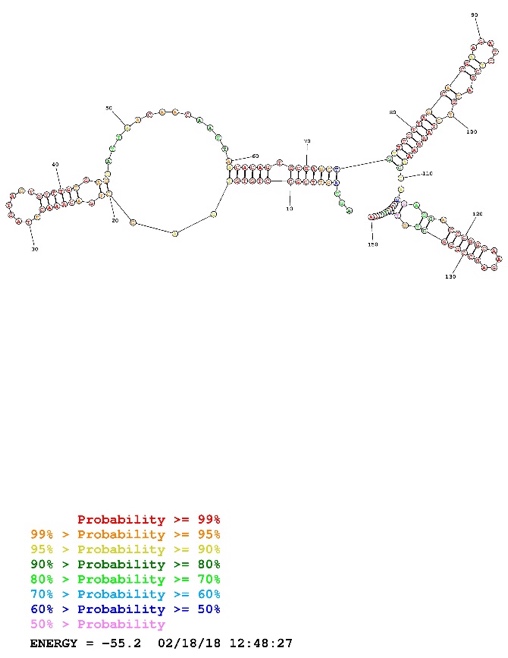  Minor | 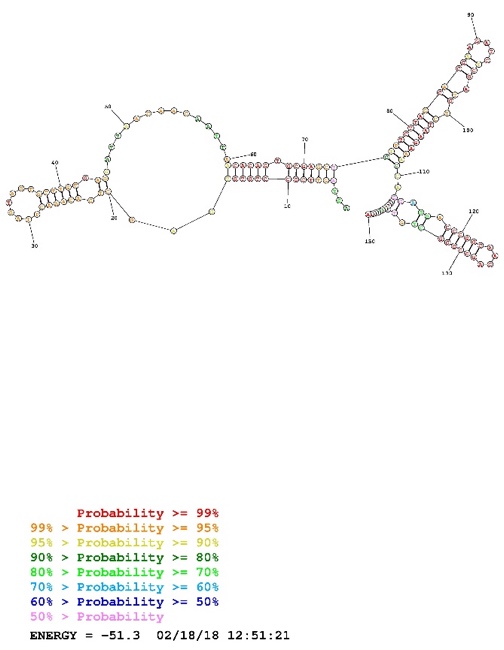  Minor |
| G121T | 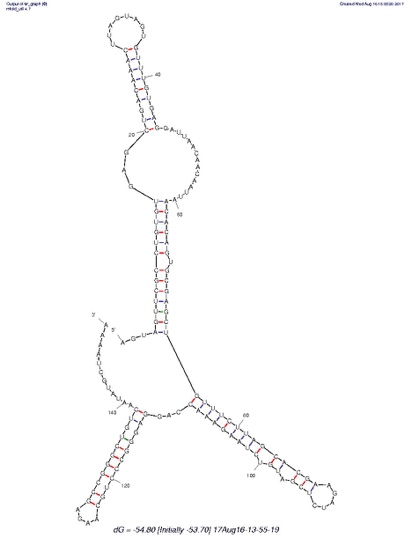  Minor | 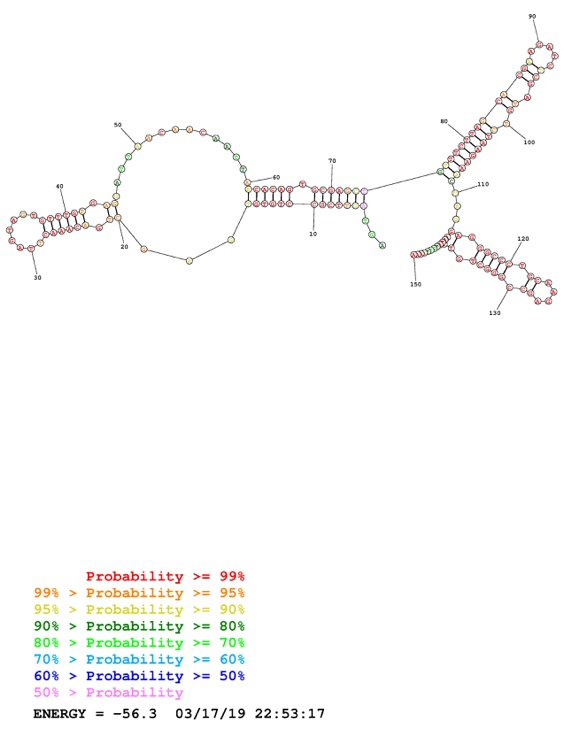  Minor | 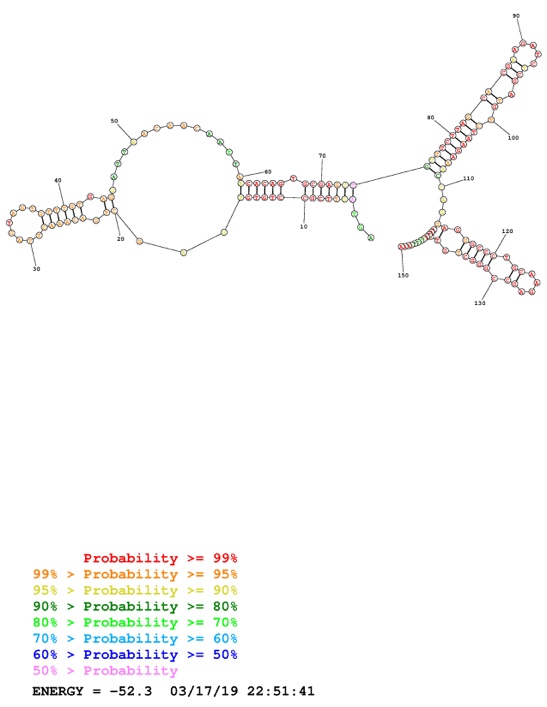  Minor |
| G126T | 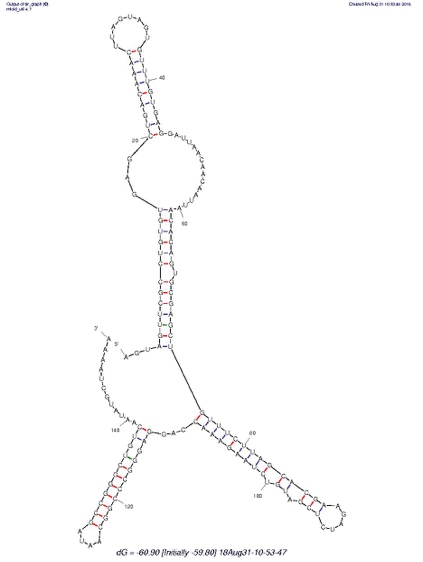  None | 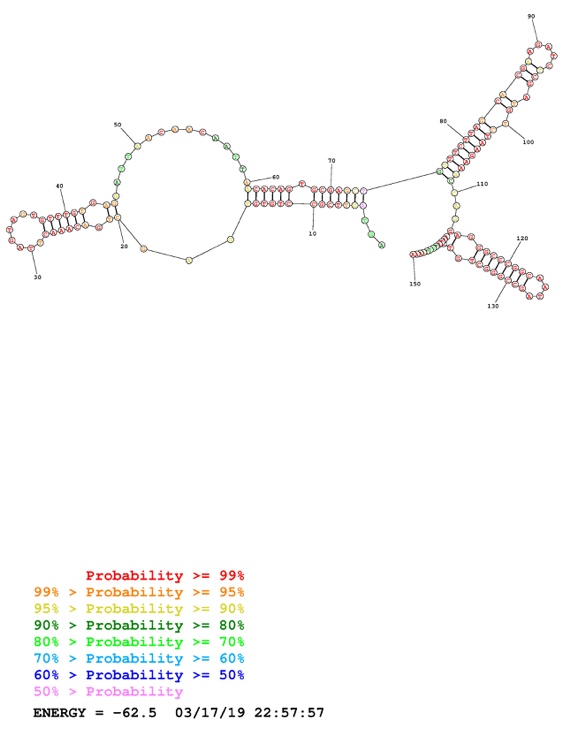  None | 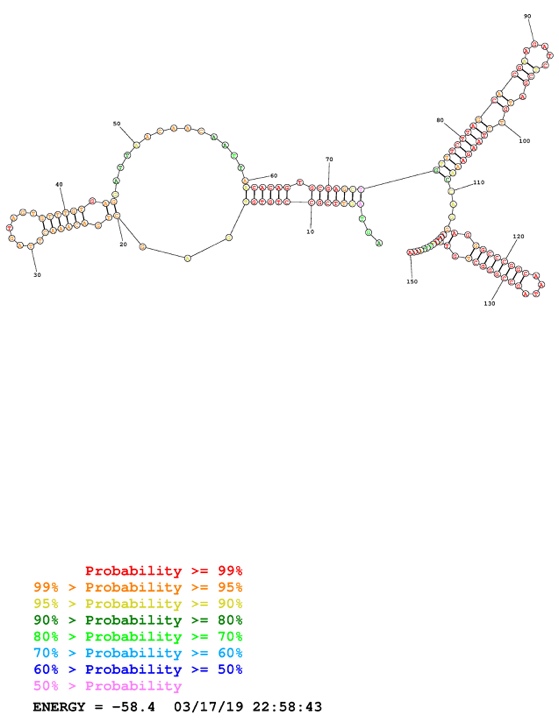  None |
| T3A | 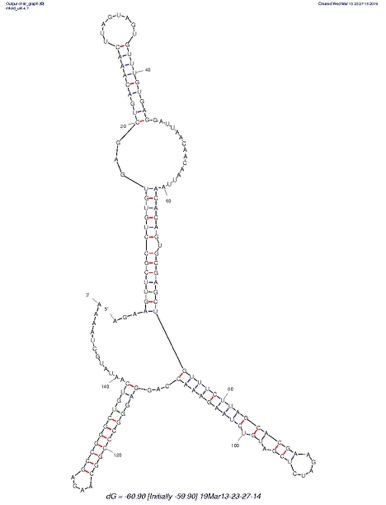  None | 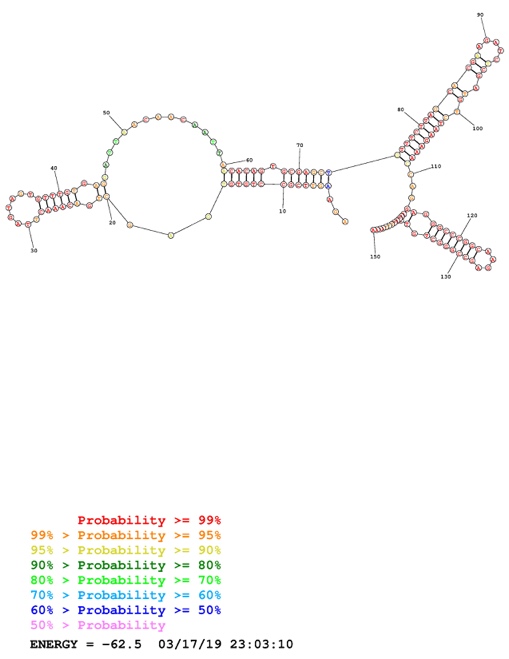  None | 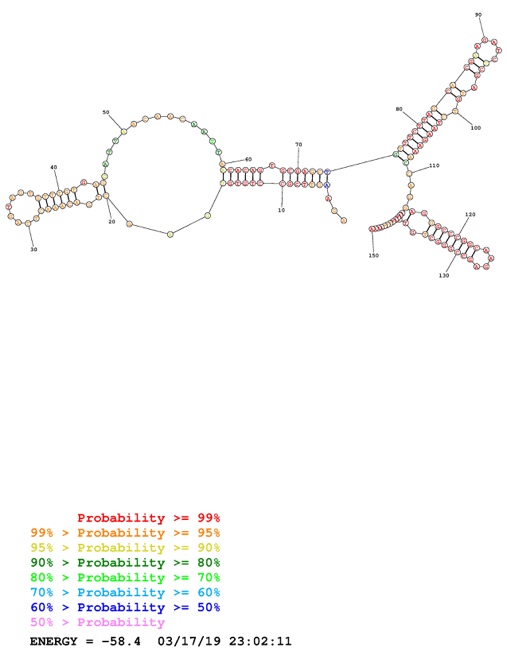  None |
| T58C | 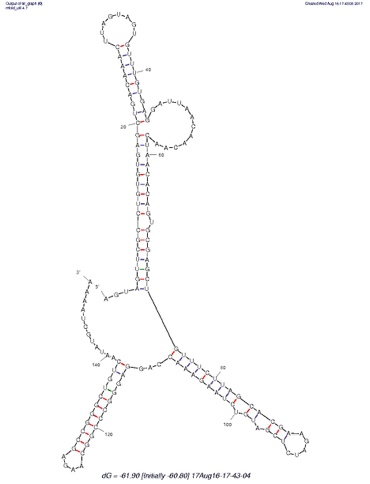  Minor | 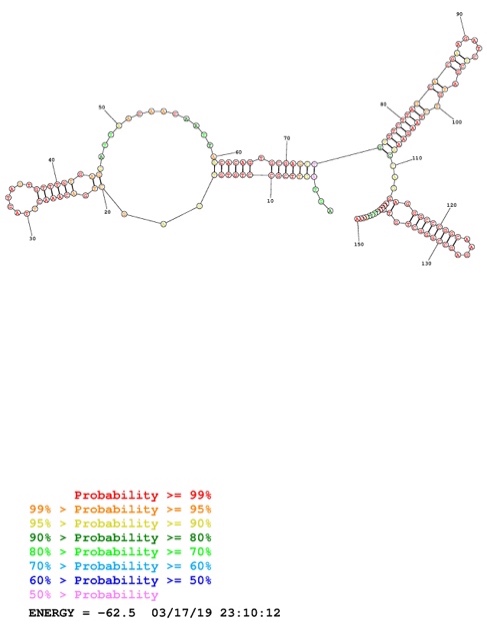  None | 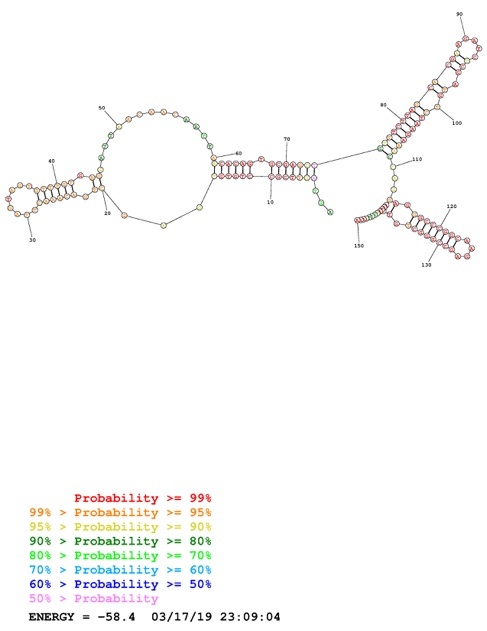  None |
