# Supplementary material for: Endless Forms: Within-Host Variation in the Structure of the West Nile Virus RNA Genome during Serial Passage in Bird Hosts
Source: mSphere. 2019 Jun 26;4(3):e00291-19. doi: 10.1128/mSphere.00291-19 (PMC6595145; doi:10.1128/mSphere.00291-19)
Supplement: TABLE S3 [file mSphere.00291-19-st003.docx]

|  | mFold First Structure | mFold Second Structure |
| --- | --- | --- |
| Wildtype | 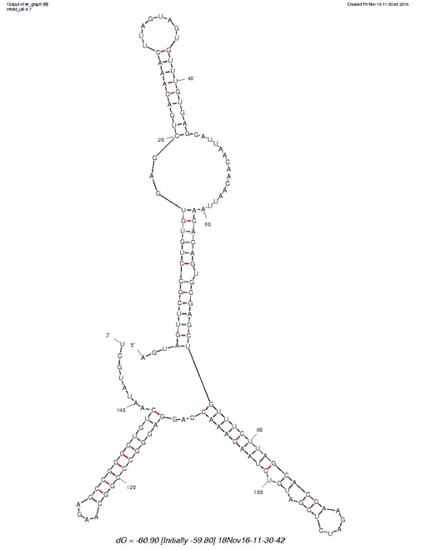  (-59.80) | 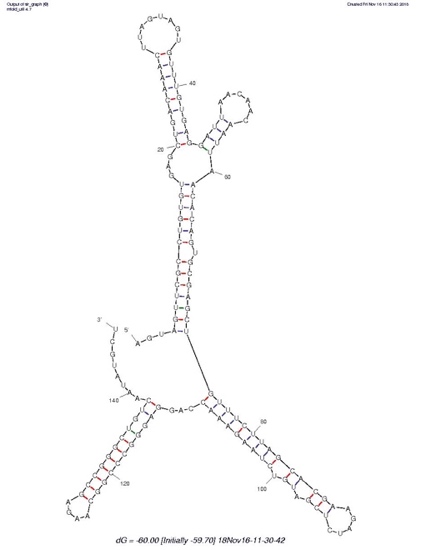  (-59.70) |
| A26T | 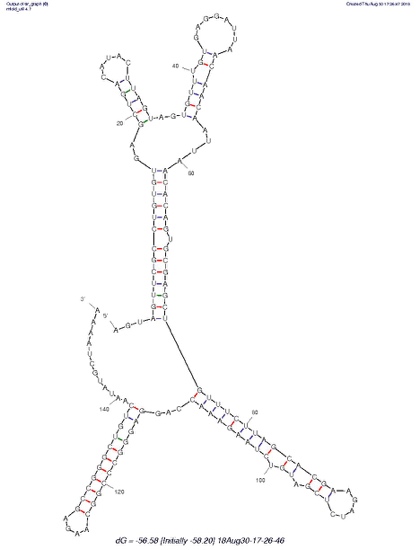  Major (-58.20) | 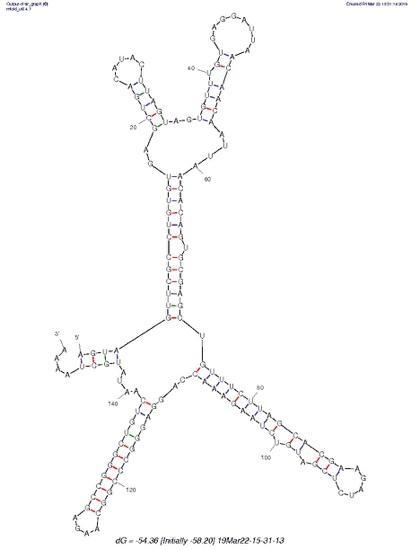  Major (-58.20) |
| A34T | 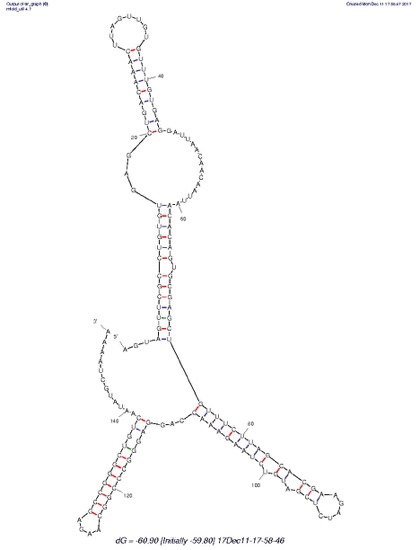  None (-59.80) | 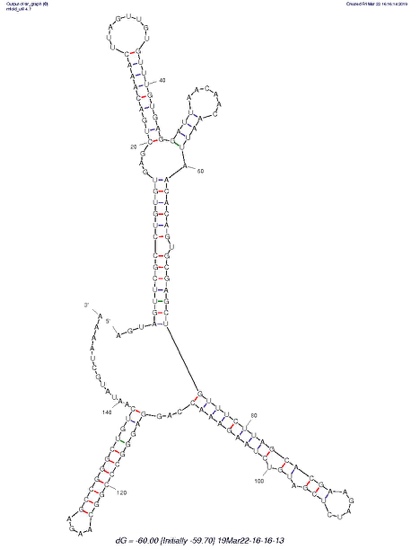  None (59.80) |
| A50G | 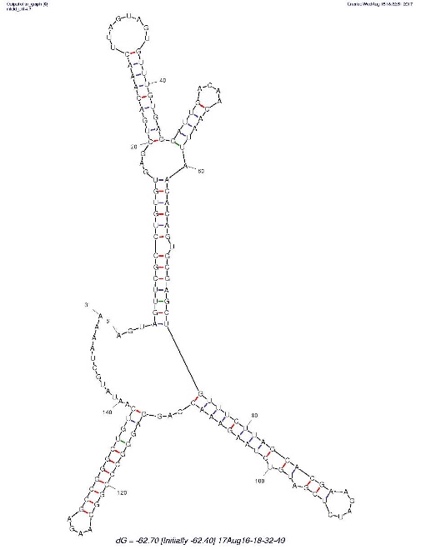  Major (-62.40) | Only 1 structure predicted |
| A65G | 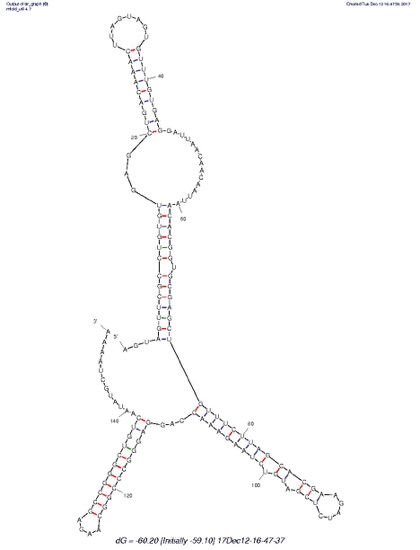  None (-59.10) | 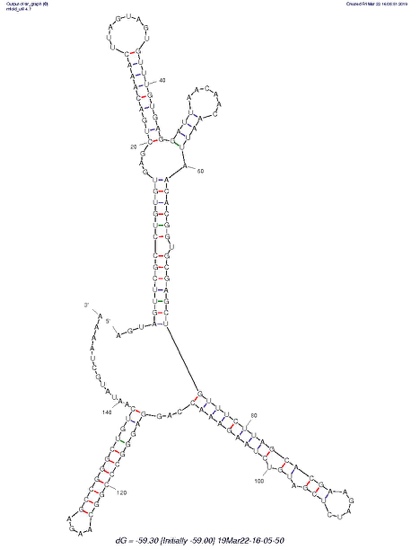  None (-59.00) |
| A106G | 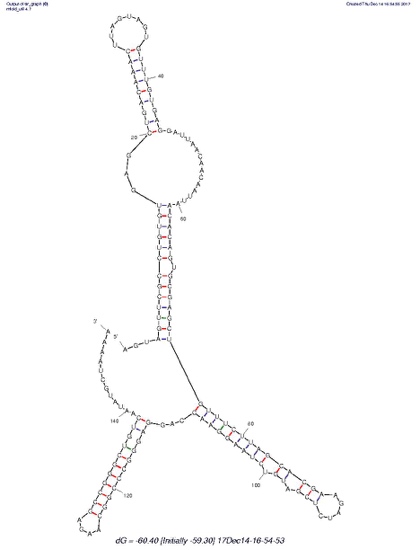  None (-59.30) | 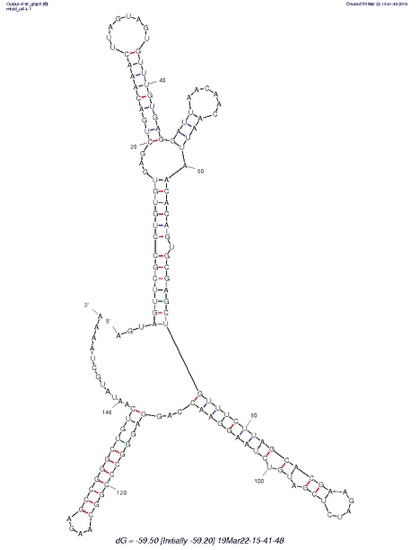  None (-59.20) |
| A111T | 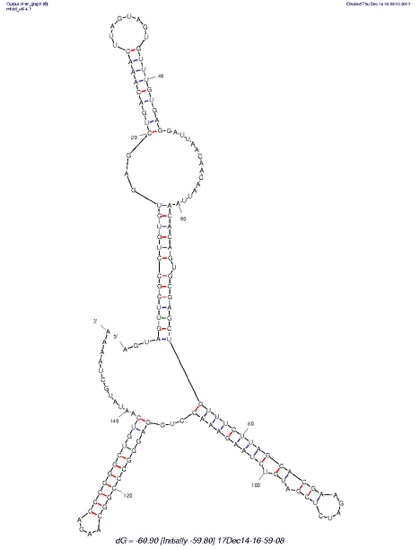  None (-59.80) | 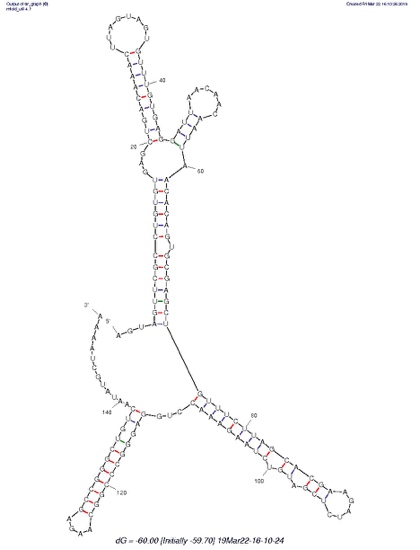  None (-59.70) |
| A127T | 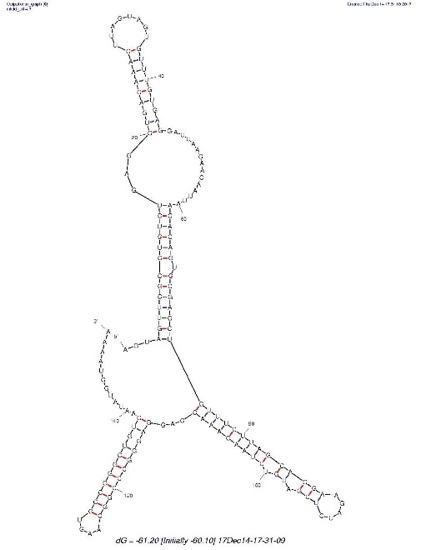  None (-60.10) | 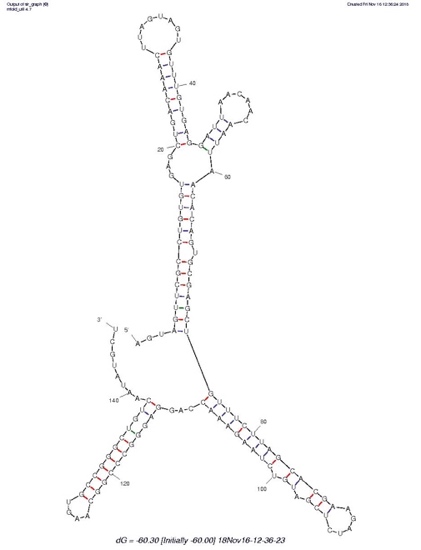  None (-60.00) |
| C10T | 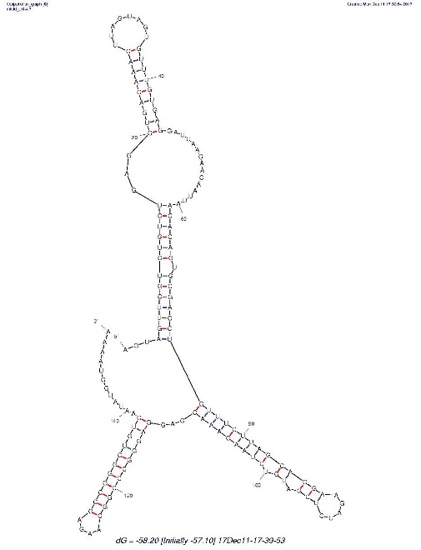  None (-57.10) | 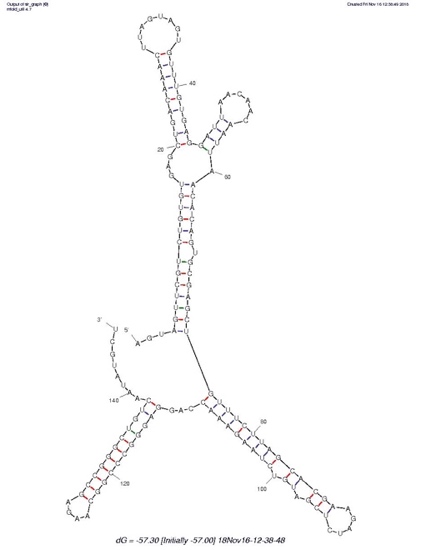  None (-57.00) |
| C118A | 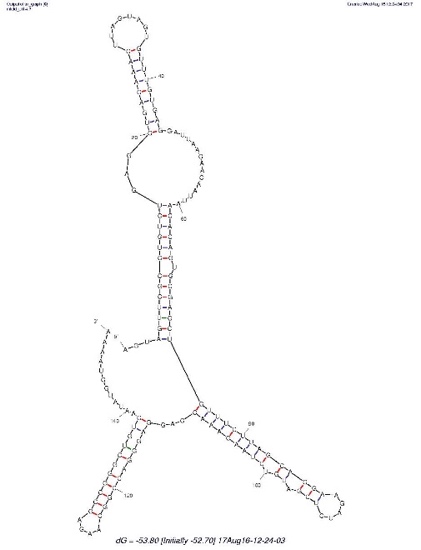  Minor (-52.70) | 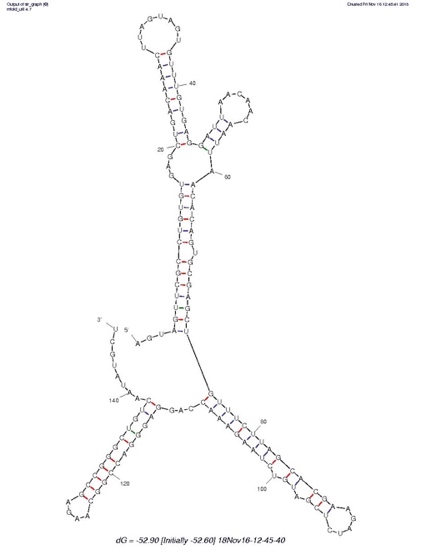  Minor (-52.60) |
| C119G | 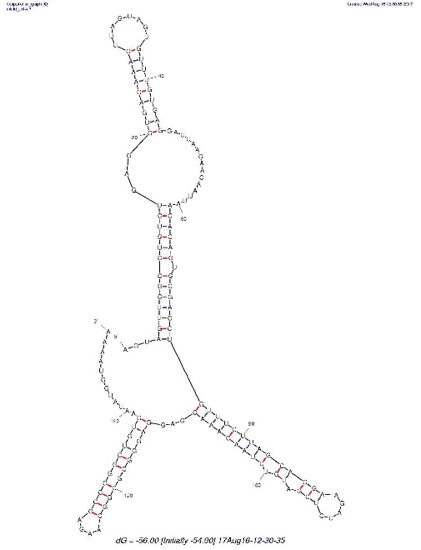  Minor (-54.90) | 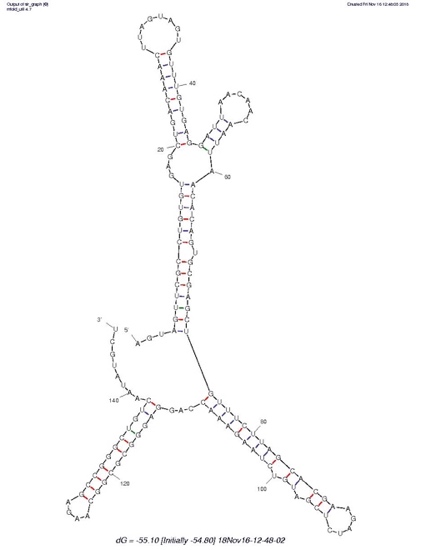  Minor (-54.80) |
| G115A | 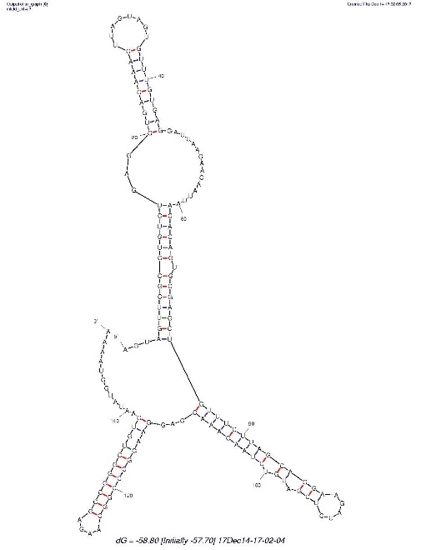  None (-57.70) | 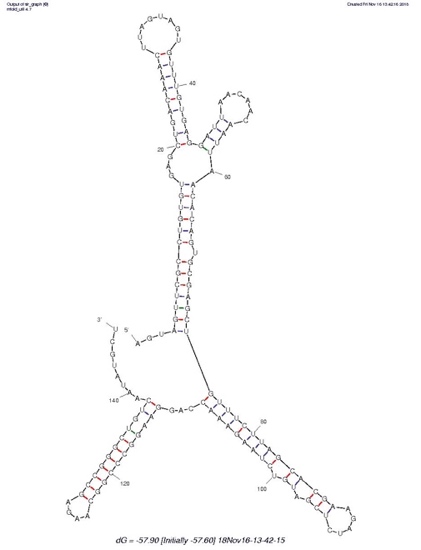  None (-57.60) |
| G117A | 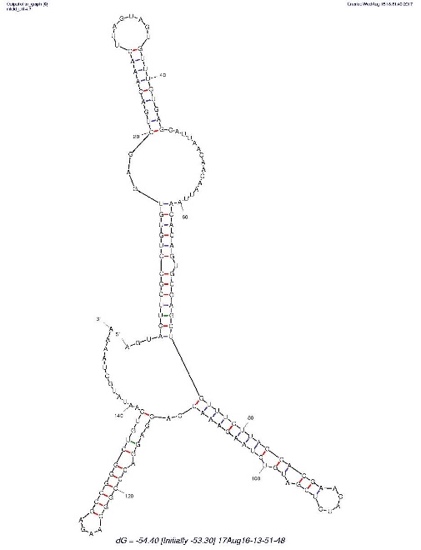  Major (-53.30) | 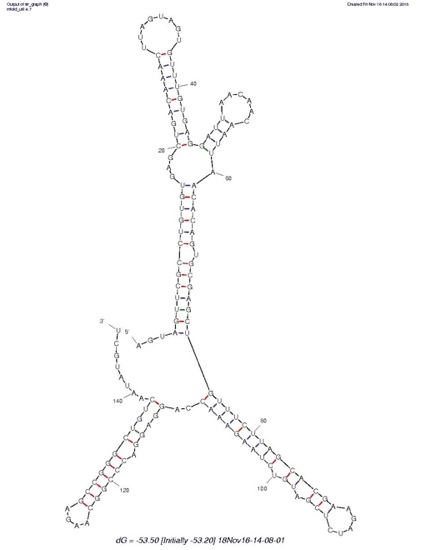  Major (-53.20) |
| G121T | 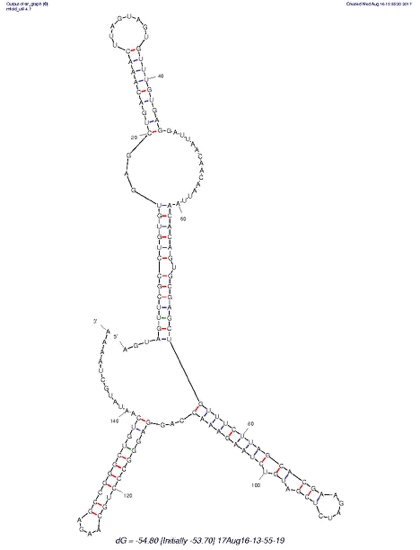  Minor (-53.70) | 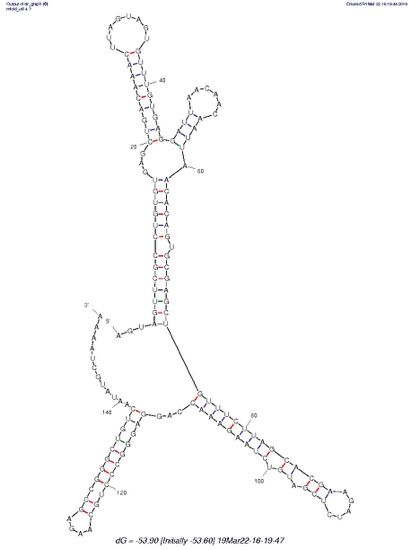  Minor (-53.60) |
| G126T | 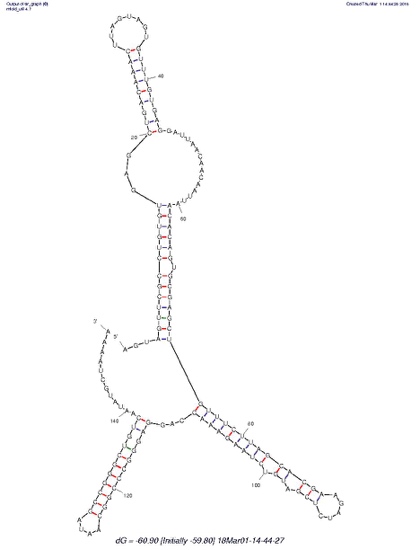  None (-59.80) | 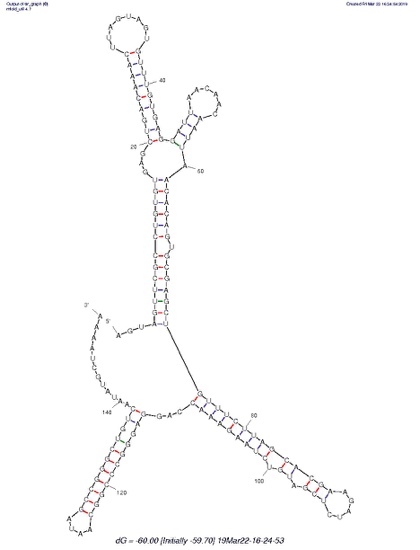  None (-59.70) |
| T3A | 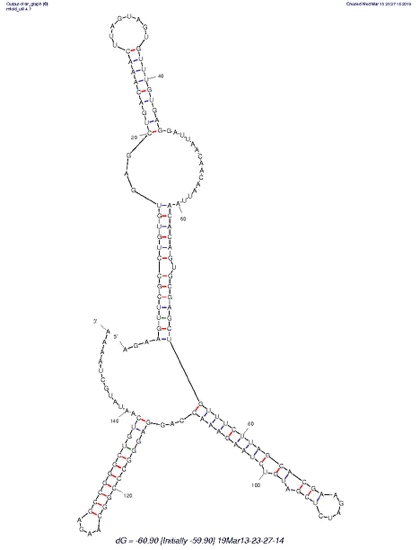  None (-59.90) | 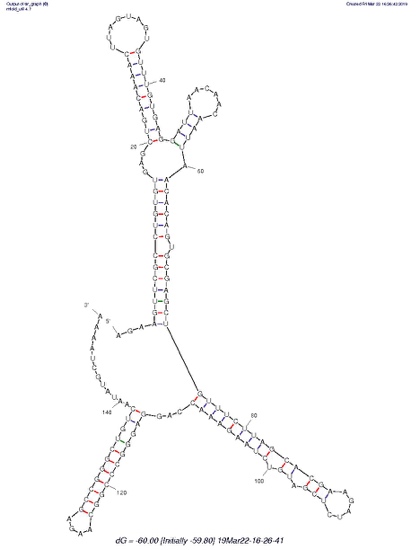  None (-59.80) |
| T58C | 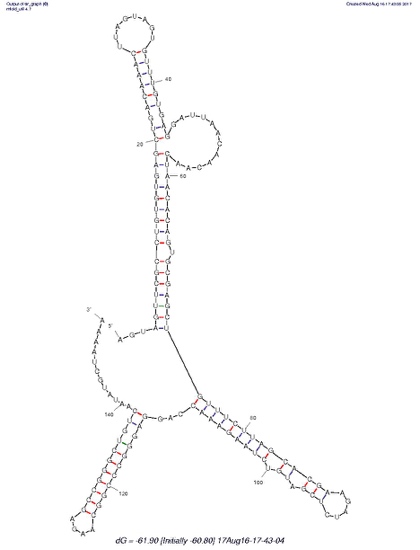  Minor (-60.80) | 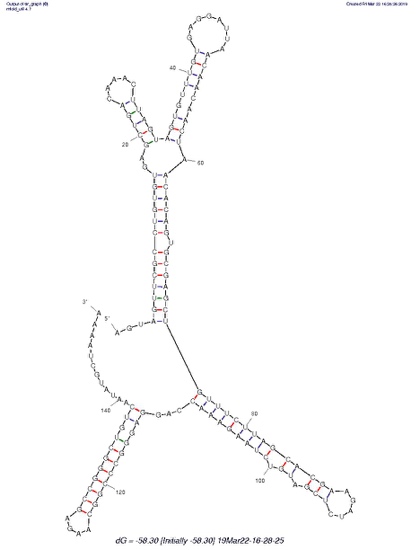  Major (-58.30) |
