# Supplementary material for: Endless Forms: Within-Host Variation in the Structure of the West Nile Virus RNA Genome during Serial Passage in Bird Hosts
Source: mSphere. 2019 Jun 26;4(3):e00291-19. doi: 10.1128/mSphere.00291-19 (PMC6595145; doi:10.1128/mSphere.00291-19)
Supplement: TABLE S4 [file mSphere.00291-19-st004.docx]

|  | **Crow** | | | **Sparrow** | | | **Robin** | | |
| --- | --- | --- | --- | --- | --- | --- | --- | --- | --- |
| **Passage** | **χ^2^ (df)** | **P** | **Adj. P** | **χ^2^ (df)** | **P** | **Adj. P** | **χ^2^ (df)** | **P** | **Adj. P** |
| 1 | 7.3 (1) | 0.007 | 0.02 | 0.2 (1) | 0.7 | 1.0 | 1.5 (1) | 0.3 | 0.9 |
| 3 | 0.4 (1) | 0.5 | 1.0 | 0.01 (1) | 0.9 | 1.0 | 0.1 (1) | 0.7 | 1.0 |
| 5 | 0.2 (1) | 0.7 | 1.0 | 2.1 (1) | 0.1 | 0.3 | 2.8 (1) | 0.1 | 0.3 |
