# Supplementary material for: Endless Forms: Within-Host Variation in the Structure of the West Nile Virus RNA Genome during Serial Passage in Bird Hosts
Source: mSphere. 2019 Jun 26;4(3):e00291-19. doi: 10.1128/mSphere.00291-19 (PMC6595145; doi:10.1128/mSphere.00291-19)
Supplement: TABLE S5 [file mSphere.00291-19-st005.docx]

|  | **Crow** | | **Sparrow** | | **Robin** | |
| --- | --- | --- | --- | --- | --- | --- |
| **Passage** | **χ^2^ (df)** | **P** | **χ^2^ (df)** | **P** | **χ^2^ (df)** | **P** |
| 1 | 5.4 (4) | 0.2 | 4.1 (4) | 0.4 | 25.6 (4) | 0.0001 |
| 3 | 10.6 (4) | 0.03 | 7.6 (4) | 0.1 | 11.2 (4) | 0.02 |
| 5 | 11.3 (4) | 0.02 | 11.1 (4) | 0.03 | 9.7 (4) | 0.05 |
